# Modeling biomarker variability in joint analysis of longitudinal and time-to-event data

**DOI:** 10.1093/biostatistics/kxad009

**Published:** 2023-05-25

**Authors:** Chunyu Wang, Jiaming Shen, Christiana Charalambous, Jianxin Pan

**Affiliations:** Department of Mathematics, The University of Manchester, Manchester, M13 9PL, UK; MRC Biostatistics Unit, University of Cambridge, Cambridge, CB2 0SR, UK; Department of Mathematics, The University of Manchester, Manchester, M13 9PL, UK; Department of Mathematics, The University of Manchester, Manchester, M13 9PL, UK; Research Center for Mathematics, Beijing Normal University, Zhuhai, China; Guangdong Provincial Key Laboratory of Interdisciplinary Research and Application for Data Science, BNU-HKBU United International College, Zhuhai, China

**Keywords:** Fully exponential Laplace approximation, Joint modeling, MRC trial, Splines, Variability

## Abstract

The role of visit-to-visit variability of a biomarker in predicting related disease has been recognized in medical science. Existing measures of biological variability are criticized for being entangled with random variability resulted from measurement error or being unreliable due to limited measurements per individual. In this article, we propose a new measure to quantify the biological variability of a biomarker by evaluating the fluctuation of each individual-specific trajectory behind longitudinal measurements. Given a mixed-effects model for longitudinal data with the mean function over time specified by cubic splines, our proposed variability measure can be mathematically expressed as a quadratic form of random effects. A Cox model is assumed for time-to-event data by incorporating the defined variability as well as the current level of the underlying longitudinal trajectory as covariates, which, together with the longitudinal model, constitutes the joint modeling framework in this article. Asymptotic properties of maximum likelihood estimators are established for the present joint model. Estimation is implemented via an Expectation-Maximization (EM) algorithm with fully exponential Laplace approximation used in E-step to reduce the computation burden due to the increase of the random effects dimension. Simulation studies are conducted to reveal the advantage of the proposed method over the two-stage method, as well as a simpler joint modeling approach which does not take into account biomarker variability. Finally, we apply our model to investigate the effect of systolic blood pressure variability on cardiovascular events in the Medical Research Council elderly trial, which is also the motivating example for this article.

## 1 Introduction

Our research is motivated by the Medical Research Council (MRC) elderly trial in the United Kingdom which compared diuretic and *β*-blocker regimens versus placebo, in hypertensive patients aged 65–74 years. The clinical interest lies in the effects of active treatments as well as the impact of systolic blood pressure (SBP) profile on cardiovascular risk. Although it is widely believed in medicine that the underlying usual SBP is of great importance in predicting cardiovascular disease, more and more research has recently provided insight into the role of SBP variability. Instead of dismissing SBP variability as a random fluctuation, Howard and Rothwell (2009) showed that visit-to-visit variability of SBP is reproducible within individuals over time. That is, individuals with high (low) visit-to-visit variability at one time are more likely to possess high (low) variability at another time. This non-random nature of SBP variability is crucial as it warranted the exploration on the prognostic value of visit-to-visit variability. A pioneering piece of research on this topic was conducted by Rothwell *and others* (2010 *b*), in which the prognostic significance of visit-to-visit SBP variability was reliably established in predicting stroke and other vascular events. Their conclusion that SBP variability was a strong predictor, even stronger than mean SBP for the cohorts they studied, challenged the widespread belief (the usual blood-pressure hypothesis; Rothwell, 2010) in medicine and justified the incorporation of visit-to-visit SBP variability when analyzing cardiovascular disease. However, existing measures of SBP variability in clinical research (Yano, 2017), e.g., standard deviation (SD), coefficient of variation, and successive variation, are calculated from observed SBP measurements on a person-by-person basis, which are susceptible to the number of measurements involved in calculations. In fact, these sample-based measures tend to provide unreliable estimates of variability if they are calculated from a small number of measurements (Howard and Rothwell, 2009). Further, the association between visit-to-visit variability, when quantified by above measures, and the risk of disease might be underestimated due to regression dilution (Clarke *and others*, 1999).

To deal with issues involved in naive variability measures, a mixed-effects location scale model was proposed (Lyles *and others*, 1999), where a random within-individual variance was introduced to characterize the individual-specific variability of the longitudinal biomarker. Meanwhile, the error-free variability was incorporated as a predictor in a survival submodel to assess its effect on the event of interest. Unlike the case in sample-based variability measures, this modeling framework (i) uses the true within-individual variance rather than its estimated version as a predictor to avoid the regression dilution issue; (ii) allows borrowing strength across individuals by assuming that these within-individual variances come from a common population (Barrett *and others*, 2019; Gao *and others*, 2011). However, this modeling approach cannot distinguish the biological (systematic) variability with which we are concerned, from the measurement variability (fluctuation due to measurement errors) since the within-individual variance is a combination of both kinds of variability. If a complicated mean function is specified to capture the biomarker trend, the within-individual variance will be dominated by measurement variability. Therefore, the degree to which random within-individual variance reflects real biological variability is uncertain.

**Fig. 1:**
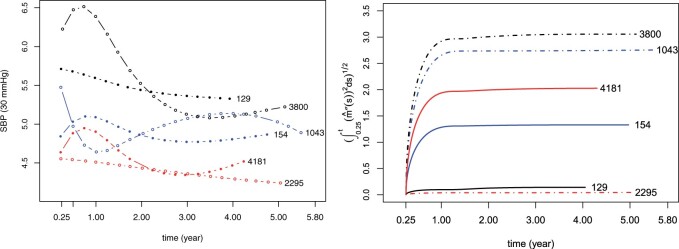
Fitted SBP trajectories (left) and the corresponding square root of cumulative variability (right). Numbers beside curves indicate patient IDs in the MRC trial. Solid circles (solid lines in the right) represent uncensored cases, while hollow circles (dashed lines in the right) represent censored cases.

In this article, we propose a measure to characterize the inherent biological variability of a biomarker whose underlying trajectory possesses smooth and nonlinear shape. The idea comes from smoothing splines on quantifying the roughness of a curve. Specifically, we use the integrated squared second derivative, $\int_{t_{0}}^{t}{\left\{m_{i}^{\prime \prime }(s)\right\}}^{2}ds$, to capture the cumulative variability of a biomarker trajectory $m_{i}(\cdot )$ for the *i*-th individual from *t*_0_ to current time *t*. The square root of the integral rather than the integral itself is used as a covariate in the survival submodel to ensure that the scale of our defined variability measure is consistent with that of $m_{i}(t)$. Figure 1 shows the fitted SBP trajectories (left, divided by 30) for six randomly selected patients in the MRC trial and their corresponding square root of cumulative variability (right). It is obvious that individuals with stable SBP level, e.g., individual 129 and 2295, have lower variability quantified by our proposed measure and that individuals with sharp fluctuation in SBP trajectory, e.g., individual 3800 and 1043, possess larger variability. From this perspective, it is reasonable to adopt this measure to characterize the biological variability behind the longitudinal measurements for each individual.

Together with a mixed-effects model for longitudinal measurements, a survival hazard model which incorporates the proposed variability quantity as a predictor, constitutes the joint modeling framework in this article. Random effects are employed in both the mixed-effects model and the survival model to account for the dependence between the two types of outcomes. Based on the standard joint model which associates longitudinal and event outcomes through the current true level of longitudinal biomarker (see Tsiatis and Davidian, 2004; Tsiatis *and others*, 1995), several extensions have been proposed to characterize possibly more complex associations. Ye *and others* (2008) included the current slope of the biomarker trajectory when modeling survival hazard. Sylvestre and Abrahamowicz (2009) incorporated the integral of the longitudinal trajectory, representing the cumulative effects of longitudinal history. All of these extended associations share a common parameterization that they can be simplified to linear functions of random effects. Clearly, the association structure in our joint model is complicated by the inclusion of the proposed variability quantity and falls outside the linear forms. For estimation, the likelihood method is the most widely used approach in joint modeling literature (Hsieh *and others*, 2006). Allowing the baseline function to be totally unspecified, Zeng and Cai (2005) established the asymptotic properties for the semiparametric maximum likelihood estimator (MLE) in which the association structures were constrained to be linear forms of random effects. We here extend their theoretical result to the joint model with our derived association structure.

The remainder of this article is organized as follows. We illustrate the motivation and rationale of quantifying the biomarker variability by our proposed measure in Section 2. In Section sec:mod, we describe the joint modeling framework with longitudinal and survival submodels specified in detail. Section 4 gives the asymptotic properties of maximum likelihood estimates (MLEs) in the proposed joint model. Section 5 focuses on the technical issues in estimation when the expectation-maximization (EM) algorithm is applied, especially on the approximation method in the E-step. In Section 6, we compare the proposed joint modeling method with other existing approaches via simulation studies. An application of our methodology to analyze the MRC trial is presented in Section 7, followed by discussion in Section 8.

## 2 Motivation

**Fig. 2:**
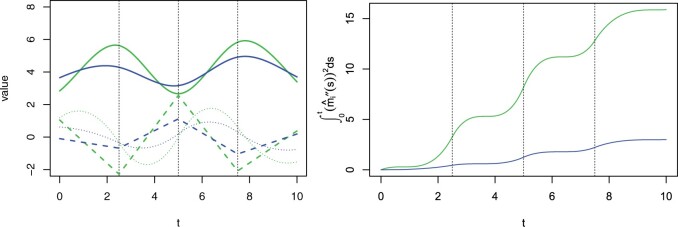
Left: simulated trajectories $m_{i}(t)$ for *i* = 1, 2 (solid curves) and their first (dotted curves) and second (dashed lines) derivative functions. Right: values of $\int_{0}^{t}m_{i}^{\prime \prime }{\left(s\right)}^{2}ds$ over time for each trajectory. The dashed vertical lines in both subfigures denote the positions of interior knots.

In the context of smoothing splines and penalized splines, a roughness penalty is imposed to control the smoothness of the estimated regression function:

(2.1) $$\sum_{i=1}^{m}{\left\{Y_{i}-m\left(t_{i}\right)\right\}}^{2}+\lambda \int_{a}^{b}m^{\prime \prime }{\left(t\right)}^{2}dt,$$

where ${\left\{(t_{i},Y_{i})\right\}}_{i=1}^{m}$ are observations and *m*(*t*) is an unknown regression function. For smoothing splines, *m*(*t*) is almost totally unspecified with the only constraint that *m*(*t*) is a twice continuously differentiable function on [a,b], i.e., $m(t)\in C^{2}[a,b]$. The minimizer of (2.1) in $C^{2}[a,b]$ is exactly a natural cubic spline with knots in all unique *t_i_*’s. For penalized splines, *m*(*t*) is prespecified by spline basis functions whose dimension is relatively large. The inclusion of the roughness penalty prevents overfitting even though a large number of knots are positioned in both contexts. By writing (2.1) in a matrix form

$$||Y-B\beta |{|}_{2}^{2}+\lambda ||J\beta |{|}_{2}^{2},$$

where *B* is the design matrix determined by the spline bases for *m*(*t*) and *J* is a matrix such that $||J\beta |{|}_{2}^{2}=\int_{a}^{b}m^{\prime \prime }{\left(t\right)}^{2}dt$, we can see that the penalty term definitely acts on the estimate of the regression coefficients *β*. Specifically, a small *λ* results in an estimate of *β* which closely fits the data (for smoothing splines, the estimate of *β*, when *λ* = 0, yields a regression function exactly interpolating all the observations), and a large *λ* leads to an estimate of *β* which corresponds to a smooth (less wiggly) fitted regression function. In other words, the penalty term $\int_{a}^{b}m^{\prime \prime }{\left(t\right)}^{2}dt$, which characterizes the roughness (the degree of fluctuation) nature of *m*(*t*) over [a,b], is determined by *β* given fixed knots of basis functions. This inspired us to use a $\int_{a}^{b}m_{i}^{\prime \prime }{\left(t\right)}^{2}dt$-based quantity to measure the variability of the subject-specific biomarker trajectory $m_{i}(t)$. We illustrate our idea through a toy example as shown in Figure 2. Two trajectories (solid curves) are specified by the same knot sequence but different vectors of regression coefficients which result in different degrees of fluctuation. This difference, as demonstrated in the right figure, can be successfully captured by $\int_{0}^{t}m_{i}^{\prime \prime }{\left(s\right)}^{2}ds$, which can be interpreted as the cumulative variability of $m_{i}(\cdot )$ up to time *t*.

## 3 Model

Let $T_{i}^{*}$ and *C_i_* denote the event and censoring times, respectively, for the *i*th individual, i=1,…,m. We can only observe $T_{i}=\min\{T_{i}^{*},C_{i}\}$ and $\delta_{i}=I\{T_{i}^{*}\leq C_{i}\}$ in practice, where I(·) is the indicator function. The longitudinal measurements for the *i*th individual are denoted by $Y_{i}={\left(Y_{i1},\ldots ,Y_{in_{i}}\right)}^{⊤}$, which are intermittently collected with measurement errors at time points $\left(t_{i1},\ldots ,t_{in_{i}}\right)$. Moreover, we denote the hypothetical trajectory behind *Y_i_* by $Y_{i}^{*}(t)$, which, in this article, is assumed to be a smooth function of *t*. It is also assumed that *C_i_* is independent of $T_{i}^{*}$ given covariates.

### 3.1 Longitudinal submodel

**Fig. 3:**
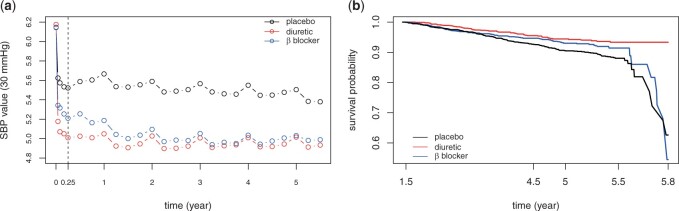
Preliminary analysis of SBP observations and cardiovascular events. (a) mean level of SBP observations (divided by 30) by randomized trials. (b) Kaplan-Meier survival curves of cardiovascular events by randomized trials.

Suppose that the underlying trajectory behind the observed longitudinal measurements is a function which depends on baseline covariates linearly and on time nonparametrically, then we specify the following semiparametric model for $Y_{i}^{*}(t)$,

(3.1) $$Y_{i}^{*}(t)=x_{i}^{⊤}\eta +m_{i}(t),\;i=1,\ldots ,m,$$

where *η* is the coefficient vector of baseline covariates, $m_{i}(t)$ is an unknown smooth function of time and might take somewhat different shapes for different individuals. Usually, the observed longitudinal value *Y_ij_* is assumed to be $Y_{i}^{*}(t_{ij})$ plus random error. Here, to accommodate the features of SBP measurements in the MRC trial (see Figure 3(a)), we allow *Y_ij_* to be affected by some other covariates *z_ij_*, which, in the MRC trial, is an indicator of whether the *j*th SBP was measured by a doctor. So we consider the following model

(3.2) $$Y_{ij}=Y_{i}^{*}(t_{ij})+z_{ij}^{⊤}\xi +\varepsilon_{ij},$$

where $\varepsilon_{ij}\overset{\mathrm{iid}}{∼}N(0,\sigma^{2})$ is the random error and *ξ* is the regression coefficient of the outside stimulus *z_ij_*. To characterize the subject-specific trajectories and the within-subject correlation, we model $m_{i}(t)$ using regression splines with multidimensional random effects,

(3.3) $$m_{i}(t)=\sum_{k=1}^{q}\left(\beta_{k}+b_{ik})B_{k}(t\right),$$

where ${\left\{B_{k}(\cdot )\right\}}_{k=1}^{q}$ is a *q*-dimensional cubic B-spline basis on follow-up time [0,τ] with a fixed knot sequence; $\beta ={\left(\beta_{1},\ldots ,\beta_{q}\right)}^{⊤}$ is a vector of fixed effects and $b_{i}={\left(b_{i1},\ldots ,b_{iq}\right)}^{⊤}$ is a vector of random effects for the *i*th individual. It is assumed that *b_i_*’s are independent and identically distributed with a common distribution $N_{q}(0,D)$ and that the *b_i_*’s are also independent with the $\varepsilon_{i}$’s. Intuitively, ${\left\{m_{i}(t)\right\}}_{i=1}^{m}$ is a collection of random trajectories varying around a common mean trend $\sum_{k=1}^{q}\beta_{k}B_{k}(t)$ with random deviations specified by random effects. Moreover, (3.1) and (3.3) suggest that baseline covariates *x_i_* only affect the intercept of $Y_{i}^{*}(t)$. In fact, we can flexibly allow *x_i_* to affect the linear trend of the trajectory by decomposing the space of B-spline basis functions into a linear plane and its orthogonal space. To this end, a proper linear transformation is required to construct the modified B-spline basis functions which are orthogonal to the linear space from the original spline bases in (3.3). But for simplicity, we mainly focus on the longitudinal submodel specified by (3.1), (3.2) and (3.3) in subsequent sections.

Note that (3.3) is a sieve approximation (Rice and Wu, 2001) to the unknown function of time. The performance of (3.3) depends on the choice for the number of knots and their locations. A pragmatic approach is to place interior knots at locations where there is high curvature in longitudinal responses and where more observations are collected, and then use AIC or BIC (calculated on longitudinal data only) to determine the number of knots (Rizopoulos *and others*, 2009; Shi *and others*, 1996).

### 3.2 Survival submodel

Motivated by the MRC trial, we allow the event time to depend on the true trajectory $Y_{i}^{*}(t)$ of longitudinal biomarker and also on its cumulative variability quantified with our proposed measure $\int_{t_{0}}^{t}{\left\{m_{i}^{\prime \prime }(s)\right\}}^{2}ds$. Therefore, the survival model is specified as

(3.4) $$\lambda_{i}(t)=\lambda_{0}(t)\;\text{exp}\;\left\{\gamma^{⊤}w_{i}+\alpha_{1}\{x_{i}^{⊤}\eta +m_{i}(t)\}+\alpha_{2}{\left(\int_{t_{0}}^{t}{\left\{m_{i}^{\prime \prime }(s)\right\}}^{2}ds\right)}^{1/2}\right\},t>t_{0}$$

where $\lambda_{i}(t)=\underset{\Delta t\to 0}{\lim}\frac{1}{\Delta t}P\left(t\leq T_{i}^{⋆}<t+\Delta t|T_{i}^{⋆}\geq t\right)$ is the survival hazard at time *t*, $\lambda_{0}(\cdot )$ is the unspecified baseline hazard function, *γ* is a coefficient vector of baseline covariates *w_i_*, *α*_1_, and *α*_2_ are parameters characterizing the effects of the current hypothetical biomarker level and the square root of cumulative variability. The lower limit of the integral, namely *t*_0_, is pre-specified in practice. Usually, *t*_0_ is set as 0 if we are interested in the cumulative variability from the start of the study to the current time. However, as is the case with the MRC trial in Section 7, *t*_0_ can be a time point other than 0 to exclude the early stage after treatment initiation. In this case ($t_{0}>0$), (3.4) is not applicable to modeling the survival hazard at $t<t_{0}$ and we assume individuals are not at risk before *t*_0_.

Under the formulation of $m_{i}(t)$ in (3.3), the integral $\int_{t_{0}}^{t}{\left\{m_{i}^{\prime \prime }(s)\right\}}^{2}ds$ involved in (3.4) can be further simplified. Recall the definition of B-splines via Cox-de Boor recursion formula:

$$B_{k,1}(s)=\{\begin{array}{cc} 1 & \;\text{if}\;s_{k}\leq s<s_{k+1},\\ 0 & \text{otherwise}; \end{array}$$

and

$$B_{k,l}(s)=\frac{s-s_{k}}{s_{k+l-1}-s_{k}}B_{k,l-1}(s)+\frac{s_{k+l}-s}{s_{k+l}-s_{k+1}}B_{k+1,l-1}(s),\;l>1;$$

where $B_{k,l}(s)$ is the *k*th B-spline basis function of order *l* (degree *l* – 1) for the given knot sequence $\left\{s_{1},\ldots ,s_{k},\ldots \right\}$. In particular, the cubic B-spline functions in (3.3) is exactly $B_{k,4}(s)$. Let ${\tilde{b}}_{i}=\beta +b_{i}$, a *q*-dimensional mixed effect vector and $\tilde{B}(s)={\left(B_{3,2}(s),\ldots ,B_{q,2}(s)\right)}^{⊤}$, a (q−2)-dimensional vector consisting of linear B-spline basis functions. A straightforward calculation gives

(3.5) $$\begin{array}{cc} \int_{t_{0}}^{t}{\left\{m_{i}^{\prime \prime }(s)\right\}}^{2}ds & ={\tilde{b}}_{i}^{⊤}Q\left(\int_{t_{0}}^{t}\tilde{B}(s){\tilde{B}}^{⊤}(s)ds\right)Q^{⊤}{\tilde{b}}_{i}=:{\tilde{b}}_{i}^{⊤}QR(t_{0},t)Q^{⊤}{\tilde{b}}_{i}, \end{array}$$

where *Q* is a q×(q−2) matrix with entries *Q_ij_*, for i=1,…,q and j=2,…,q−1, given by

$$Q_{j-1,j}=\frac{6}{\left(s_{j+3}-s_{j+1})(s_{j+3}-s_{j}\right)},\;\;Q_{j+1,j}=\frac{6}{\left(s_{j+3}-s_{j+1})(s_{j+4}-s_{j+1}\right)},$$

with $Q_{jj}=-(Q_{j-1,j}+Q_{j+1,j})$ and *Q_ij_* = 0 for |i−j|≥2; $R(t_{0},t)$ is a (q−2)×(q−2) symmetric matrix with elements $R_{ij}(t_{0},t)$, for *i* and *j* running from 2 to (q−1), given by

$$R_{ii}(t_{0},t)=\int_{t_{0}}^{t}{\left\{B_{i+1,2}(s)\right\}}^{2}ds,\;R_{i,i+1}(t_{0},t)=R_{i+1,i}(t)=\int_{t_{0}}^{t}B_{i+1,2}(s)B_{i+2,2}(s)ds,$$

with $R_{ij}(t_{0},t)=0$ for |i−j|≥2 as $B_{k_{1},2}(s)$ and $B_{k_{2},2}(s)$ are nonoverlapping when $|k_{1}-k_{2}|\geq 2$ (de Boor, 1978).

Let $K(t_{0},t)=QR(t_{0},t)Q^{⊤}$. Substituting (3.5) into (3.4) gives the following expression for the survival hazard

(3.6) $$\lambda_{i}(t)=\lambda_{0}(t)\;\text{exp}\;\left[\gamma^{⊤}w_{i}+\alpha_{1}\{x_{i}^{⊤}\eta +{\tilde{b}}_{i}^{⊤}B(t)\}+\alpha_{2}{\left\{{\tilde{b}}_{i}^{⊤}K(t_{0},t){\tilde{b}}_{i}\right\}}^{1/2}\right],$$

where $B(t)={\left(B_{1}(t),\ldots ,B_{q}(t)\right)}^{⊤}$. As ${\tilde{b}}_{i}^{⊤}K(t_{0},t){\tilde{b}}_{i}\leq \lambda_{\text{max}}||{\tilde{b}}_{i}|{|}^{2}$ with $\lambda_{\text{max}}$ denoting the maximum eigenvalue of the positive semidefinite matrix $K(t_{0},t),\;{\left({\tilde{b}}_{i}^{⊤}K(t_{0},t){\tilde{b}}_{i}\right)}^{1/2}$ is therefore of order $O({\tilde{b}}_{i})$, the same as that of $m_{i}(t)$. This is the reason we use ${\left({\tilde{b}}_{i}^{⊤}K(t_{0},t){\tilde{b}}_{i}\right)}^{1/2}$ instead of ${\tilde{b}}_{i}^{⊤}K(t_{0},t){\tilde{b}}_{i}$ in the survival hazard model.

## 4 Inference on semiparametric model

Let $\theta ={\left(\gamma^{⊤},\alpha_{1},\alpha_{2},\beta^{⊤},\eta^{⊤},\xi^{⊤},\sigma^{2},\text{Vec}{\left(D\right)}^{⊤}\right)}^{⊤}$ be the collection of unknown parameters to be estimated. Under the standard assumption that *T_i_* and *Y_i_* are conditionally independent given random effects *b_i_*, the specification of submodels (3.2) and (3.6) leads to the following likelihood:

(4.1) $$L(\theta ,\lambda_{0})=\prod_{i=1}^{m}\int \lambda_{i}{\left(T_{i}|b_{i};\theta ,\lambda_{0}\right)}^{\delta_{i}}S\left(T_{i}|b_{i};\theta ,\lambda_{0}\right)p\left(Y_{i}|b_{i};\theta \right)p\left(b_{i};\theta \right)db_{i},$$

where $S(t|b_{i})=\text{exp}\;\left\{-\int_{0}^{t}\lambda_{i}(s|b_{i})ds\right\}$ is the survival function.

Let $\Lambda_{0}(t)=\int_{0}^{t}\lambda_{0}(s)ds$ denote the cumulative baseline hazard. The likelihood (4.1) can be arbitrarily large by letting $\lambda_{0}(t)$ rise higher and higher at points close to the observed event times and vanish otherwise (Aalen *and others*, 2008, pp. 204–205). To circumvent the issue, Riemann–Stieltjes integral is employed with $\Lambda_{0}(t)$ being the integrator which is constrained to be a non-negative and nondecreasing function. It’s easy to verify that the likelihood is maximized if $\Lambda_{0}(t)$ is a step function with jumps at the observed event times $\left\{T_{i}:\delta_{i}=1\right\}$. So instead of maximizing $L(\theta ,\lambda_{0})$ directly, we work on the modified likelihood function (Zeng and Cai, 2005):

(4.2) $$\begin{array}{c} L_{m}(\theta ,\Lambda_{0})\propto \prod_{i=1}^{m}\int {\left(\Lambda_{0}\{T_{i}\}\;\text{exp}\;\left[\gamma^{⊤}w_{i}+\alpha_{1}\left\{x_{i}^{⊤}\eta +{\tilde{b}}_{i}^{⊤}B(T_{i})\right\}+\alpha_{2}{\left\{{\tilde{b}}_{i}^{⊤}K(t_{0},T_{i}){\tilde{b}}_{i}\right\}}^{1/2}\right]\right)}^{\delta_{i}}\\ \;\times \text{exp}\;\left(-\int_{t_{0}}^{T_{i}}\;\text{exp}\;\left[\gamma^{⊤}w_{i}+\alpha_{1}\left\{x_{i}^{⊤}\eta +{\tilde{b}}_{i}^{⊤}B(t)\right\}+\alpha_{2}{\left\{{\tilde{b}}_{i}^{⊤}K(t_{0},t){\tilde{b}}_{i}\right\}}^{1/2}\right]d\Lambda_{0}(t)\right)\\ \;\times {\left(\sigma^{2}\right)}^{-n_{i}/2}\;\text{exp}\;\left(-\frac{1}{2\sigma^{2}}{\left\|Y_{i}-X_{i}\eta -B_{i}{\tilde{b}}_{i}-Z_{i}\xi \right\|}_{2}^{2}\right)|D{|}^{-1/2}\;\text{exp}\;\left(-\frac{1}{2}b_{i}^{⊤}D^{-1}b_{i}\right)db_{i}, \end{array}$$

where $\Lambda_{0}\{T_{i}\}$ is the jump size of $\Lambda_{0}(\cdot )$ at time *T_i_*; *X_i_*, *Z_i_* and $B_{i}$ are design matrices corresponding to baseline covariates (*X_i_* is constructed by repeating *x_i_* for *n_i_* times), external stimuli and B-spline bases with $B_{i}={\left(B(t_{i1}),\ldots ,B(t_{in_{i}})\right)}^{⊤}$.

The maximum likelihood estimate $\left(\hat{\theta },{\hat{\Lambda }}_{0}\right)$ is defined as $\left(\hat{\theta },{\hat{\Lambda }}_{0})=\arg\max_{\theta \in \Theta ,\Lambda_{0}\in Z_{m}}\;\text{log}\;L_{m}(\theta ,\Lambda_{0}\right)$, where $Z_{m}$ consists of all right-continuous step functions only with positive jumps at $\left\{T_{i}:\delta_{i}=1\right\}$. Let $\theta^{*}$ denote the true parameter for θ and $\Lambda_{0}^{*}(\cdot )$ be the true cumulative baseline function. Under assumptions given in Section SA of the supplementary material available at *Biostatistics* online, we establish the consistency and asymptotic normality of $\left(\hat{\theta },{\hat{\Lambda }}_{0}\right)$. Moreover, by showing the influence function (each coordinate) of $\hat{\theta }$ falls into a linear span of score functions, $\hat{\theta }$ is proved to be semiparametrically efficient.

Theorem 4.1 (Consistency). Under conditions specified in Section SA of the supplementary material available at *Biostatistics* online, the MLE $\left(\hat{\theta },{\hat{\Lambda }}_{0}\right)$ is strongly consistent:

$$||\hat{\theta }-\theta^{*}|{|}_{2}+||{\hat{\Lambda }}_{0}-\Lambda_{0}^{*}|{|}_{\infty }\to 0,\;a.s.$$

where $||\cdot |{|}_{2}$ is the Euclidean norm and $||\cdot |{|}_{\infty }$ is the supremum norm on [0,τ].

Let $l^{\infty }[0,\tau ]$ be the metric space of all bounded functions in [0,τ] and let *d* denote the dimension of θ. The next theorem states the asymptotic distribution of $\left(\hat{\theta },{\hat{\Lambda }}_{0}\right)$.

Theorem 4.2 (Asymptotic normality and efficiency). Under conditions specified in Section SA of the supplementary material available at *Biostatistics* online, $\sqrt{m}\left(\hat{\theta }-\theta^{*},{\hat{\Lambda }}_{0}-\Lambda_{0}^{*}\right)$ converges in distribution to a Gaussian random element in $R^{d}\times l^{\infty }[0,\tau ]$. Further, $\sqrt{m}\left(\hat{\theta }-\theta^{*}\right)$ weakly converges to a multivariate normal distribution with mean zero and variance equal to the semiparametric efficiency bound.

Proofs of the above theorems are similar in spirit to Zeng and Cai (2005) and can be found in Section SA of the supplementary material available at *Biostatistics* online. As the analytical form for the asymptotic covariance matrix is not available (Hsieh *and others*, 2006), we employ a boostrap method to numerically estimate the standard errors of $\hat{\theta }$. Basically, we generate *J* bootstrap samples (each of size *m*) by sampling with replacement from the original observed data and estimate $\left(\theta ,\Lambda_{0}\right)$ based on each bootstrap sample. The SD of *J* bootstrap estimates of θ is then an estimate of the standard error of $\hat{\theta }$.

Remark 4.3 The asymptotic theorems developed in this section aim to extend the asymptotic properties of the semiparametric MLE in standard join models (with a linear association structure in terms of random effects) to our proposed joint models with a complex association structure in (3.6). However, we should note that (3.3) is an approximation (a regression spline estimator) to an unknown smooth trajectory and the consequence of this approximation behavior has not been taken into account in Theorems 4.1 and 4.2. Consistency of the regression spline estimator definitely requires the dimension of spline bases *q* increasing to infinity at an appropriate rate of *m* (*q* should be written as *q_m_*). See Agarwal and Studden (1980) for the order of *q_m_* under which the regression spline estimator and its derivatives achieve the optimal global convergence rate. Similar to regression dilution with error-prone covariates, it is expected that the survival parameters, especially *α*_1_ and *α*_2_, will be estimated with bias. We illustrate this phenomenon in Section 6.3 where the true biomarker trajectory is set to be a smooth function but not exactly a spline function given by (3.3).

## 5 Estimation with EM algorithm

The MLEs in the joint modeling framework can be typically obtained using the EM algorithm which is implemented by iteratively applying the E-step and M-step, with random effects treated as missing data. For the likelihood given by (4.2), the E-step refers to the calculation of

$$Q(\theta ,\Lambda_{0}|\theta^{\left(k\right)},\Lambda_{0}^{\left(k\right)})=\sum_{i=1}^{m}\int [\;\text{log}\;p\left(T_{i},\delta_{i},Y_{i},b_{i};\theta ,\Lambda_{0}\right)]p\left(b_{i}|T_{i},\delta_{i},Y_{i};\theta^{\left(k\right)},\Lambda_{0}^{\left(k\right)}\right)db_{i},$$

where $\left(\theta^{\left(k\right)},\Lambda_{0}^{\left(k\right)}\right)$ is the estimated value of $\left(\theta ,\Lambda_{0}\right)$ obtained from iteration *k*, and the M-step refers to the maximization of $Q(\theta ,\Lambda_{0}|\theta^{\left(k\right)},\Lambda_{0}^{\left(k\right)})$ with respect to $\left(\theta ,\Lambda_{0}\right)$.

### 5.1 M-step

Let $E_{i(k)}[\cdot ]$ and $V_{i(k)}[\cdot ]$ denote the abbreviations of $E[\cdot |T_{i},\delta_{i},Y_{i};\theta^{\left(k\right)},\Lambda_{0}^{\left(k\right)}]$ and $\text{Var}[\cdot |T_{i},\delta_{i},Y_{i};\theta^{\left(k\right)},\Lambda_{0}^{\left(k\right)}]$, respectively, i.e., the expectation and variance with respect to the conditional distribution of *b_i_* given $\left(T_{i},\delta_{i},Y_{i}\right)$ and the current value $\left(\theta^{\left(k\right)},\Lambda_{0}^{\left(k\right)}\right)$ of parameters. Denote $N=\sum_{i=1}^{m}n_{i}$ as the total number of longitudinal measurements. It is not difficult to find that some components of $\left(\theta ,\Lambda_{0}),\;\phi_{2}:=(\xi ,\sigma^{2},D,\Lambda_{0}\right)$, can be updated in closed forms. That is, $\phi_{2}^{\left(k+1\right)}=f(\phi_{1}^{\left(k+1\right)}|\theta^{\left(k\right)},\Lambda_{0}^{\left(k\right)})$ for some function f(·), where $\phi_{1}=(\gamma ,\alpha_{1},\alpha_{2},\eta ,\beta )$ are components with no closed updating form. Specifically, the jump size of $\Lambda_{0}(\cdot )$ at time point *t* is updated by

$$\begin{array}{c} \Lambda_{0}^{\left(k+1\right)}\{t\}=\left\{\sum_{i=1}^{m}\delta_{i}I\left(T_{i}=t\right)\right\}/\sum_{i=1}^{m}I(T_{i}\geq t)E_{i(k)}\left[r(b_{i},t,\phi_{1}^{\left(k+1\right)})\right] \end{array},$$

where $r(b_{i},t,\phi_{1})=\text{exp}\;\left[\gamma^{⊤}w_{i}+\alpha_{1}\{x_{i}^{⊤}\eta +{\tilde{b}}_{i}^{⊤}B(t)\}+\alpha_{2}{\left\{{\tilde{b}}_{i}^{⊤}K(t_{0},t){\tilde{b}}_{i}\right\}}^{1/2}\right]$ is the relative risk for the *i*th individual at time *t*. The specific updating forms for $\xi ,\sigma^{2}$ and *D* are presented in Section SB of the supplementary material available at *Biostatistics* online. For $\phi_{1}$, the update $\phi_{1}^{\left(k+1\right)}$ is a solution to the equation $S_{\phi_{1}}:=\partial Q(\theta ,\Lambda_{0}|\theta^{\left(k\right)},\Lambda_{0}^{\left(k\right)})/\partial \phi_{1}{|}_{(\phi_{1},\phi_{2})=\left(\phi_{1},f(\phi_{1}|\theta^{\left(k\right)},\Lambda_{0}^{\left(k\right)})\right)}=0$. As no closed-form solution is available, we resort to a one-step Newton–Raphson method in which the updating at the (k+1)th iteration is performed as $\phi_{1}^{\left(k+1\right)}=\phi_{1}^{\left(k\right)}+\nu_{k}I_{\phi_{1}^{\left(k\right)}}^{-1}S_{\phi_{1}^{\left(k\right)}}$ with the step size *ν_k_* determined by backtracking line search. For $I_{\phi_{1}^{\left(k\right)}},-\partial S_{\phi_{1}}/\partial \phi_{1}{|}_{\phi_{1}=\phi_{1}^{\left(k\right)}}$ is used when updating $\gamma ,\alpha_{1}$, and *α*_2_, while $\sum_{i=1}^{n}S_{i\phi_{1}^{\left(k\right)}}S_{i\phi_{1}^{\left(k\right)}}^{⊤}$ is recommended when updating *β* and *η* so as to avoid complicated calculations involved in calculating second derivatives (Griffiths *and others*, 1987).

### 5.2 E-step

It is worth noting that the expectations involved in the M-step are of the following form

(5.1) $$\begin{array}{c} E_{i(k)}\left[A(b_{i})\right]=\int A\left(b_{i}\right)p\left(b_{i}|T_{i},\delta_{i},Y_{i};\theta^{\left(k\right)},\Lambda_{0}^{\left(k\right)}\right)db_{i}=\frac{\int A\left(b_{i}\right)p\left(T_{i},\delta_{i},Y_{i},b_{i};\theta^{\left(k\right)},\Lambda_{0}^{\left(k\right)}\right)db_{i}}{\int p\left(T_{i},\delta_{i},Y_{i},b_{i};\theta^{\left(k\right)},\Lambda_{0}^{\left(k\right)}\right)db_{i}}, \end{array}$$

where $A(b_{i})$ denotes a general function of *b_i_*, which may take the forms in Section SB of the supplementary material available at *Biostatistics* online. In view of the highly nonlinear shape of the longitudinal subject-specific trajectories, the random effects introduced in (3.3) are usually not of low dimension, which makes calculating the above integrals a challenge as the (adaptive) Gauss-Hermite method suffers from the curse of dimensionality. To balance the computation burden and approximation accuracy, we use a fully exponential Laplace approximation method. The idea behind this method is to apply the standard Laplace method in both the numerator and denominator of (5.1), which makes the $O\left(n_{i}^{-1}\right)$ terms canceled and leads to an $O\left(n_{i}^{-2}\right)$ approximation (Rizopoulos *and others*, 2009; Tierney *and others*, 1989). Specifically, under the fully exponential Laplace approximation, integrals in form (5.1) can be calculated as follows

(5.2) $$\begin{array}{c} E\left[A\left(b_{i}\right)|T_{i},\delta_{i},Y_{i};\theta^{\left(k\right)},\Lambda_{0}^{\left(k\right)}\right]=A\left({\hat{b}}_{i}\right)-\frac{1}{2}\text{tr}\left({I_{i}^{-1}\left\{\partial I_{i}^{\left(c\right)}/\partial c^{T}\right\}|}_{(c,b)=\left(0,{\hat{b}}_{i}\right)}\right)+O\left(n_{i}^{-2}\right), \end{array}$$

where ${\hat{b}}_{i}=\text{arg}\max_{b_{i}}\;\text{log}\;p\left(T_{i},\delta_{i},Y_{i},b_{i};\theta^{\left(k\right)},\Lambda_{0}^{\left(k\right)}\right)$ and $I_{i}=I_{i}^{\left(c\right)}{|}_{(c,b)=\left(0,{\hat{b}}_{i}\right)}$ with

$$I_{i}^{\left(c\right)}=-\frac{\partial^{2}[\;\;\text{log}\;\;p\left(T_{i},\delta_{i},Y_{i},b_{i};\theta^{\left(k\right)},\Lambda_{0}^{\left(k\right)}\right)+c^{⊤}A(b_{i})]}{\partial b_{i}^{⊤}\partial b_{i}}.$$

As indicated in (5.2), only the mode of $\text{log}\;p\left(T_{i},\delta_{i},Y_{i},b_{i};\theta^{\left(k\right)},\Lambda_{0}^{\left(k\right)}\right)$, namely ${\hat{b}}_{i}$, and second derivatives of $\text{log}\;p\left(T_{i},\delta_{i},Y_{i},b_{i};\theta^{\left(k\right)},\Lambda_{0}^{\left(k\right)}\right)$ and $A(b_{i})$ around ${\hat{b}}_{i}$ are required to achieve a second order approximation of $E_{i(k)}\left[A(b_{i})\right]$. Given that in the present model ${\hat{b}}_{i}$’s cannot be obtained analytically, Newton–Raphson method is employed to find the value numerically. See Section B of the supplementary material available at *Biostatistics* online for specific calculations.

### 5.3 Impact on asymptotic properties

Note that the asymptotic properties, including consistency of $\left(\hat{\theta },{\hat{\Lambda }}_{0}\right)$ and semiparametric efficiency of $\hat{\theta }$, established in Section 4, may be affected due to approximation method used in the E-step. Based on investigations in Vonesh (1996) and Rizopoulos *and others* (2009), the resulting convergence rate of $\left(\hat{\theta },{\hat{\Lambda }}_{0}\right)$ when a fully exponential Laplace approximation is applied becomes $\left(\hat{\theta }-\theta^{*},{\hat{\Lambda }}_{0}-\Lambda_{0}^{*}\right)=O_{p}(\max\{m^{-1/2},\min{\left(n_{i}\right)}^{-2}\}),$ where $\min{\left(n_{i}\right)}^{-2}$ comes from the approximation error in the E-step. Therefore, $\left(\hat{\theta },{\hat{\Lambda }}_{0}\right)$ will be consistent only as both *m* and $n_{i},i=1,\ldots m$ go to infinity, and the consistency rate will still be $O_{p}(m^{-1/2})$ if $\min(n_{i})$ grows at a rate greater than $m^{1/4}$. Moreover, if $\min(n_{i})$ grows faster than $m^{1/2}$, the asymptotic behavior remains the same as shown in Theorem 4.2. This can be illustrated from the difference between $Q^{\mathrm{FEL}}(\theta ,\Lambda_{0}|\theta^{\left(k\right)},\Lambda_{0}^{\left(k\right)})$, the fully exponential Laplace based Q function, and $Q(\theta ,\Lambda_{0}|\theta^{\left(k\right)},\Lambda_{0}^{\left(k\right)})$, the true Q function. Since $Q^{\mathrm{FEL}}(\theta ,\Lambda_{0}|\theta^{\left(k\right)},\Lambda_{0}^{\left(k\right)})=Q(\theta ,\Lambda_{0}|\theta^{\left(k\right)},\Lambda_{0}^{\left(k\right)})+O(m\min{\left(n_{i}\right)}^{-2})$, the resulting estimate, when $O(m\min{\left(n_{i}\right)}^{-2})$ is negligible, will act as if it is calculated from the true Q function or exact likelihood function. Semiparametric efficiency of $\hat{\theta }$ still holds in this case.

## 6 Simulation studies

### 6.1 Case 1: General simulation study

We specify a longitudinal submodel with three evenly spaced interior knots (*q* = 7) within the follow-up time interval [0,10] and observation times $t_{ij}=(0,1,\ldots ,20)/2$. Regression coefficients are set as β=(6,3,7,1,8,5,4). The variance (covariance matrix) of measurement error and random effects are specified as $\sigma^{2}=1$ and D=diag(3,4,4,5,4,3,4), respectively. The baseline covariates *x_i_* and outside stimulus *z_i_* are not incorporated in the longitudinal model for simplicity. For the survival submodel, we consider a binary covariate *w* taking values 0 or 1, each with probability 1/2 and its effect is assumed to be γ=−2. The baseline hazard function $\lambda_{0}(t)$ is specified as exp {−2} if *t* > 5 and 0 otherwise. Censoring times $C_{i},i=1,\ldots ,m$ are independently generated from Exp(0.05), leading to a 50% censoring rate. Truncated by event times and censoring times, the number of longitudinal observations *n_i_* varies between 11 and 21. The simulation setting described above aims to define a simple case to numerically examine the performance of estimation methods. 500 Monte Carlo (MC) replications are conducted and the sample size is fixed as *m* = 1000.

Table 1 shows the performance of the proposed joint modeling (JM) method against the two-stage (TS) method (Wu *and others*, 2012). Both methods are evaluated in terms of bias, Monte Carlo SD, bootstrap SD $\left({\text{SD}}^{b}\right)$ and 95% coverage probability (CP). The estimates of survival parameters, namely, $\gamma ,\alpha_{1}$, and *α*_2_, show larger biases under TS than those obtained from the JM method. In particular, the effects of the longitudinal level and variability on survival hazard are underestimated when the TS method is employed. CPs for the TS estimates of *γ* and *α*_2_ suffer from severe under-coverage due to large biases. Both methods provide good performance in estimating parameters related to the longitudinal submodel. Moreover, ${\text{SD}}^{b}$s are quite close to SDs, which implies that bootstrap SD performs well in estimating the standard error of $\hat{\theta }$ obtained from both JM and TS methods and hence can be used in the real data analysis. For survival parameters, the standard errors of the JM estimates are slightly larger than those of the TS estimates. This is mainly due to the fact that the randomness of *b_i_* in the survival submodel is removed when the TS method is used. Additionally, the standard errors of the ${\hat{\beta }}_{j}$’s and ${\hat{D}}_{jj}$’s increase with index *j* since the latter elements in *β* and *D* correspond to the basis functions with support over later time intervals in which longitudinal observations are sparse.

**Table 1:** Simulation results of the JM and TS methods in case 1. Average $\hat{\theta }$ is the average value of parameter estimates in 500 MC replications; SD is the MC standard deviation of the estimates across simulated data sets; ${\text{SD}}^{b}$ is the mean of bootstrap standard deviations calculated on bootstrap samples for each MC sample; CP is the 95% coverage probability defined as the percentage of repetitions in which true value falls into the calculated confidence interval.

	JM				TS			
True	$\hat{\theta }$	SD	${\text{SD}}^{b}$	CP	$\hat{\theta }$	SD	${\text{SD}}^{b}$	CP
**Survival**								
γ=−2	–1.9786	0.1011	0.0990	0.946	–1.8218	0.0878	0.0898	0.458
$\alpha_{1}=0.2$	0.1976	0.0310	0.0303	0.940	0.1667	0.0270	0.0273	0.760
$\alpha_{2}=0.3$	0.2917	0.0301	0.0290	0.940	0.2389	0.0267	0.0256	0.364
**Longitudinal**								
$\beta_{1}=6$	5.9584	0.0674	0.0663	0.912	5.9966	0.0670	0.0663	0.932
$\beta_{2}=3$	3.0902	0.0797	0.0794	0.910	3.0124	0.0788	0.0793	0.938
$\beta_{3}=7$	6.9584	0.0803	0.0826	0.920	6.9771	0.0806	0.0824	0.942
$\beta_{4}=1$	0.9824	0.0875	0.0863	0.948	1.0385	0.0873	0.0862	0.938
$\beta_{5}=8$	7.9500	0.1140	0.1220	0.930	7.8822	0.1129	0.1205	0.820
$\beta_{6}=5$	4.9798	0.1717	0.1678	0.952	4.8884	0.1713	0.1670	0.900
$\beta_{7}=4$	4.0011	0.2238	0.2285	0.944	4.0036	0.2197	0.2259	0.942
$D_{11}=3$	3.0139	0.1819	0.1868	0.950	2.9978	0.1830	0.1868	0.950
$D_{22}=4$	4.0455	0.2518	0.2569	0.942	3.9903	0.2513	0.2553	0.948
$D_{33}=4$	3.9946	0.2418	0.2517	0.948	3.9933	0.2433	0.2537	0.944
$D_{44}=5$	4.9966	0.2720	0.2785	0.946	4.9919	0.2723	0.2782	0.948
$D_{55}=4$	3.9482	0.3825	0.3703	0.940	3.9326	0.3830	0.3691	0.944
$D_{66}=3$	2.9812	0.5057	0.5220	0.942	2.9705	0.5023	0.6196	0.946
$D_{77}=4$	3.8998	0.7397	0.7366	0.942	3.8914	0.7338	0.7324	0.946
$\sigma^{2}=1$	0.9983	0.0141	0.0142	0.940	0.9998	0.0142	0.0143	0.942

### 6.2 Case 2: Simulation study based on the MRC trial

A second simulation is conducted to investigate the performance of the proposed method in analyzing the MRC trial by generating data similar to the real data. To this end, parameters are set around their estimated values. Covariates and observation schedules for longitudinal and survival outcomes are designed to mimic the real data (see Section SC.1 of the supplementary material available at *Biostatistics* online for details). Truncated by event times and censoring times, *n_i_* varies between 10 and 26 and thus $\min(n_{i})>m^{1/4}$ with *m* = 3700. The approximation error in the E-step is expected to have little effect on the JM estimation. Before showing the final estimation result, we first illustrate the validity of the knots selection criteria mentioned in Section 3.1. By calculating AIC and BIC based on longitudinal data only, the true model can be effectively selected as indicated in Table 2.

**Table 2:** Knots selection criteria with longitudinal data only.

Number	Location	AIC	BIC
*q* = 5	0.25	97 065	97 285
	1	101 295	101 515
	1.5	102 593	102 813
*q* = 6	(0.25, 0.5)	96 273	96 529
	(0.5, 1)	96 034	96 290
	(0.25, 1)	95 657	95 913
	**(0.25, 1.5)**	**95 508**	**95 765**
*q* = 7	(0.25, 1, 1.5)	95 947	96 240

Table 3 shows the estimation results in case 2. Due to the high censoring rate, biases and standard errors increase for estimates of survival parameters compared with those in case 1. For parameters of particular interest $\left(\alpha_{1},\alpha_{2}\right)$, estimates obtained from the JM method perform slightly better than those from the TS method in terms of bias and CP, but the improvement is not as significant as that in case 1. An important reason is that the predicted random effects from the longitudinal model, ${\hat{b}}_{i}^{L}$, are close to *b_i_*, as the measurement error involved in longitudinal observations is small ($\sigma^{2}=0.1764$) in case 2.

**Table 3:** Simulation results of the JM and TS methods in case 2. Average $\hat{\theta }$ is the average value of parameter estimates in 500 MC replications; SD is the MC standard deviation of the estimates across simulated data sets; ${\text{SD}}^{b}$ is the mean of bootstrap standard deviations calculated on bootstrap samples for each MC sample; CP is the 95% coverage probability defined as the percentage of repetitions in which true value falls into the calculated confidence interval.

	JM				TS			
True	$\hat{\theta }$	SD	${\text{SD}}^{b}$	CP	$\hat{\theta }$	SD	${\text{SD}}^{b}$	CP
**Survival**								
$\gamma_{1}=-0.4$	–0.4190	0.1564	0.1549	0.948	–0.4222	0.1556	0.1537	0.936
$\gamma_{2}=-0.1$	–0.1180	0.1397	0.1417	0.944	–0.1202	0.1393	0.1410	0.942
$\gamma_{3}=0.71$	0.7195	0.1016	0.1013	0.946	0.7288	0.1015	0.1012	0.940
$\gamma_{4}=0.22$	0.2025	0.1327	0.1319	0.950	0.1994	0.1326	0.1319	0.948
$\gamma_{5}=0.36$	0.3221	0.2036	0.2138	0.946	0.3157	0.2032	0.2137	0.944
$\alpha_{1}=0.37$	0.3416	0.1441	0.1384	0.934	0.3236	0.1389	0.1332	0.920
$\alpha_{2}=0.23$	0.2037	0.0844	0.0827	0.932	0.1758	0.0845	0.0823	0.870
**Longitudinal**								
$\beta_{1}=6.2$	6.1932	0.1241	0.1242	0.946	6.1944	0.1242	0.1240	0.948
$\beta_{2}=4.5$	4.4954	0.1212	0.1221	0.946	4.4947	0.1211	0.1221	0.948
$\beta_{3}=5.0$	4.9946	0.1199	0.1223	0.940	4.9950	0.1199	0.1222	0.940
$\beta_{4}=4.7$	4.6951	0.1231	0.1226	0.944	4.6936	0.1231	0.1228	0.944
$\beta_{5}=5.0$	4.9950	0.1260	0.1264	0.944	4.9907	0.1262	0.1262	0.946
$\beta_{6}=4.5$	4.4860	0.1278	0.1276	0.946	4.4851	0.1278	0.1277	0.946
$\beta_{1}^{\left(1\right)}=6.8$	6.7988	0.1301	0.1276	0.942	6.7887	0.1301	0.1275	0.942
$\beta_{2}^{\left(1\right)}=4.2$	4.1939	0.1214	0.1217	0.940	4.1935	0.1214	0.1216	0.940
$\beta_{3}^{\left(1\right)}=4.5$	4.4935	0.1212	0.1222	0.936	4.4939	0.1212	0.1214	0.934
$\beta_{4}^{\left(1\right)}=4.1$	4.0976	0.1234	0.1240	0.948	4.0968	0.1234	0.1238	0.944
$\beta_{5}^{\left(1\right)}=4.5$	4.4911	0.1307	0.1316	0.944	4.4922	0.1308	0.1315	0.946
$\beta_{6}^{\left(1\right)}=4.2$	4.1955	0.1353	0.1357	0.948	4.1950	0.1352	0.1354	0.946
$\beta_{1}^{\left(2\right)}=6.4$	6.3959	0.1277	0.1278	0.950	6.3969	0.1278	0.1277	0.948
$\beta_{2}^{\left(2\right)}=4.5$	4.4951	0.1209	0.1218	0.934	3.0124	0.1209	0.1218	0.934
$\beta_{3}^{\left(2\right)}=4.6$	4.5925	0.1208	0.1217	0.936	4.5931	0.1208	0.1217	0.936
$\beta_{4}^{\left(2\right)}=4.1$	4.0972	0.1246	0.1243	0.940	4.0961	0.1246	0.1245	0.940
$\beta_{5}^{\left(2\right)}=4.5$	4.4901	0.1331	0.1330	0.942	4.4915	0.1332	0.1329	0.946
$\beta_{6}^{\left(2\right)}=4.3$	4.2920	0.1331	0.1335	0.936	4.2914	0.1331	0.1335	0.938
$\eta_{1}=-0.05$	–0.0495	0.0084	0.0085	0.952	–0.0496	0.0084	0.0085	0.952
$\eta_{2}=0.03$	0.0297	0.0124	0.0111	0.948	0.0297	0.0124	0.0112	0.948
$\eta_{3}=0.09$	0.0907	0.0172	0.0175	0.942	0.0908	0.0172	0.0175	0.940
ξ=0.08	0.0801	0.0042	0.0042	0.948	0.0799	0.0042	0.0042	0.978
$D_{22}=0.2$	0.1993	0.0062	0.0063	0.942	0.1995	0.0062	0.0064	0.946
$D_{33}=0.2$	0.1994	0.0070	0.0069	0.954	0.1997	0.0071	0.0069	0.958
$D_{44}=0.4$	0.3988	0.0160	0.0170	0.952	0.3993	0.0160	0.0170	0.952
$D_{55}=0.5$	0.4975	0.0268	0.0263	0.954	0.4971	0.0268	0.0264	0.950
$D_{66}=0.3$	0.2975	0.0320	0.0322	0.952	0.2976	0.0320	0.0323	0.952
$\sigma^{2}=0.1764$	0.1764	0.0010	0.0010	0.930	0.1764	0.0010	0.0010	0.930

### 6.3 Case 3: Simulation study for a nonparametric setting

In this final simulation study, we examine the performance of our proposed method (the spline based sieve approximation to a biomarker trajectory and the EM algorithm in estimation) when the true biomarker trajectory is not exactly a spline function as specified in (3.3). We set the true longitudinal trajectory for the *i*-th individual as $m_{i}(t)=\nu_{1i}\{(-1/6){\left(t-3\right)}^{3}+(t-3)\}+\nu_{2i}$ for i=1,…,1000, where $\nu_{1i}$ and $\nu_{2i}$ are independent random parameters generated from U(0.5,2) and *U*(4, 8), respectively. Obviously, $\nu_{1i}$ corresponds to the degree of fluctuation for the *i*-th trajectory and $\nu_{2i}$ is related to the biomarker level. The measurement times are specified as $t_{ij}=\{0,0.4,0.8,1.2,1.6,2,2.5,\ldots 6\}$ (every 0.5 year after 2 years) during the follow-up interval [0,6] and the measurement error is generated from $N(0,0.4^{2})$, independently of $\nu_{1i}$ and $\nu_{2i}$. The baseline covariates *x_i_* and outside stimulus *z_i_* are not incorporated for simplicity. For the survival submodel, the baseline hazard function $\lambda_{0}(t)$ is specified as exp {−3} if *t* > 2 and 0 otherwise. We generate a binary baseline covariate *w_i_* from Bernoulli distribution with probability 0.5. Censoring times *C_i_*, i=1,…,1000 are independently generated from *U*(3, 10), which, together with the end of study (*τ* = 6), lead to approximately 40% censoring. Truncated by event times and right censoring, the number of longitudinal observations *n_i_* varies between 6 and 14.

We fit the joint model given by (3.1), (3.2), (3.3) and (3.6) to the simulated data. Note that the spline regression model (3.3), in this case, is an approximation or working model. To determine the number and location of interior knots, we first specify several candidate knot patterns based on quantiles of measurement times and the follow-up interval (see Section SC.2 of the supplementary material available at *Biostatistics* online), and then select the best pattern among them via AIC and BIC, which are calculated by simply fitting the longitudinal submodel. Table 4 shows the estimation result for survival parameters obtained from different methods. In addition to JM and TS mentioned before, the performance of the true model (a benchmark) where the biomarker level and variability are specified from the true $m_{i}(t)$ as in the data generating process, and that of the standard joint model (called “No Variability” in Table 4) without biomaker variability, are also reported in Table 4. It is expected that JM and TS may not perform as well as in case 1 and case 2 due to the extra bias resulting from the approximation in (3.3). Compared with *γ* and *α*_1_, the estimation of *α*_2_, in both JM and TS, suffers from significant bias and thus under-coverage of CP. However, if the biomarker variability is removed, as implemented with the standard joint model, both *γ* and *α*_1_ are estimated with substantial bias. In this sense, taking the biomarker variability into account benefits the estimation of effects of the other predictors. Table 5 shows the estimation results when the biomarker variability is not involved in generating event times, i.e., the true value of *α*_2_ is zero. These three models produce similar estimates and the null effect of biomarker variability can be successfully detected. A more challenging case (case 4) where $m_{i}(t)$ is no longer a polynomial function but a sine function instead, is considered in Section SC.3 of the supplementary material available at *Biostatistics* online. JM and TS show very similar performance in this scenario since approximating a sine function via a spline function is the dominant source of bias.

**Table 4:** Simulation results in case 3 among 500 MC replications. SD is the MC standard deviation of the estimates across simulated data sets; CP is the 95% coverage probability.

	JM			TS			No Variability			True model		
True	$\hat{\theta }$	SD	CP	$\hat{\theta }$	SD	CP	$\hat{\theta }$	SD	CP	$\hat{\theta }$	SD	CP
γ=−1	–0.9884	0.0778	0.944	–0.9664	0.0761	0.920	–0.8983	0.0765	0.726	–0.9978	0.0760	0.948
$\alpha_{1}=0.3$	0.2918	0.0319	0.936	0.2805	0.0303	0.894	0.2260	0.0319	0.356	0.3020	0.0314	0.952
$\alpha_{2}=0.3$	0.2674	0.0301	0.720	0.2438	0.0275	0.664	–	–	–	0.3009	0.0282	0.960

**Table 5:** Simulation results in case 3 among 500 MC replications when the true value of *α*_2_ is set as zero. SD is the MC standard deviation of the estimates across simulated data sets; CP is the 95% coverage probability.

	JM			TS			No Variability			True model		
True	$\hat{\theta }$	SD	CP	$\hat{\theta }$	SD	CP	$\hat{\theta }$	SD	CP	$\hat{\theta }$	SD	CP
γ=−1	–0.9995	0.0893	0.946	–1.0013	0.0897	0.946	–1.0016	0.0914	0.946	–1.0049	0.0897	0.948
$\alpha_{1}=0.3$	0.2898	0.0392	0.926	0.2827	0.0374	0.924	0.2875	0.0382	0.924	0.3019	0.0340	0.950
$\alpha_{2}=0$	0.0022	0.0331	0.948	–0.0047	0.0345	0.950	–	–	–	0.0004	0.0305	0.952

## 7 Real-data analysis

In the MRC trial, 4396 patients aged 65–74 were randomized to receive diuretic, *β* blocker or placebo. SBP values were measured fornightly for the first month, then monthly to 3 months and every 3 months thereafter. Cardiovascular (CV) events including stroke, whether non-fatal or fatal; and myocardial infarction, whether fatal or nonfatal were recorded during the follow-up. Figure 3(a) illustrates the change in group average of SBP observations (divided by 30) over time. Several points are highlighted here. (i) The average SBP trends are different in different treatment groups and therefore a treatment-dependent longitudinal model is preferred for modeling SBP trajectories. (ii) The regular peaks, occurring at the end of each year (1, 2, 3, 4, 5 years) as well as the entry visit (0 year), correspond to the measurements made by doctors with the other measurements made by nurses. The marked increase at these time points can be explained by the “white coat” effect, a pressor response to the presence of doctors or other kind of stressful circumstances (Lever and Brennan, 1993; Party *and others*, 1992), which, in our model, is treated as an outside stimulus and is excluded from the inherent SBP trend (Carr *and others*, 2012). (iii) All groups experienced immediate drop-off after entry. The drop was steepest in the first 2-weeks, then continued, more gently, for about 3 months (0.25 year) followed by a comparatively stable stage. Therefore global polynomials of lower degree may not perform well in capturing the SBP trend over time and so cubic B-spline basis functions are used instead.

In this article, we focus on the relationship between longitudinal SBP measurements and time to the first cardiovascular event, especially on the question whether the cumulative variability of the SBP profile exhibits significant association with the CV hazard. To ensure the approximation method (in the E-step) used in our EM algorithm does not lead to non-ignorable biases, we restrict our analysis to the cohort of 3784 patients who had at least 10 SBP measurements, and 310 patients among this cohort experienced the event during the follow-up period. The longitudinal submodel for SBP observations is given below:

$$\begin{array}{c} Y_{ij}=\eta_{1}{\text{Sex}}_{i}+\eta_{2}{\text{Smoke}}_{i}+\eta_{3}{\text{Age}}_{i}+\sum_{k=1}^{q}\left(\beta_{k}+I_{\left\{{\text{tmt}}_{i}=1\right\}}\beta_{k}^{\left(1\right)}+I_{\left\{{\text{tmt}}_{i}=2\right\}}\beta_{k}^{\left(2\right)}+b_{ik}\right)B_{k}(t_{ij})\\ +\xi {\text{WC}}_{ij}+\varepsilon_{ij}≐x_{i}^{⊤}\eta +m_{i}(t_{ij})+\xi {\text{WC}}_{ij}+\varepsilon_{ij}, \end{array}$$

where $x_{i}=({\text{Sex}}_{i},{\text{Smoke}}_{i},{\text{Age}}_{i})$ is a vector of baseline covariates with ${\text{Age}}_{i}$ denoted as original age divided by 10; $m_{i}(t)$ is the subject-specific SBP trend over time with fixed effects being treatment-dependent (tmt=1 or 2 corresponds to diuretic or *β* blocker treatment); ${\text{WC}}_{ij}$, the “white coat” indicator, takes value 1 if the *j*-th measurement was measured by a doctor and $\varepsilon_{ij}∼N(0,\sigma^{2})$ is the measurement error independent of random effect *b_i_*. In light of SBP profiles (see Figure 3(a)) together with AIC and BIC criteria, we set *q* = 6 with interior knots located at 0.25 and 1.5. Preliminary analysis based on longitudinal data only (ignoring event times) shows that the variance of $b_{i1}$ is very close to zero. So $b_{i}=(b_{i2},\ldots ,b_{iq-1})∼N_{q-1}(0,D)$ with $b_{i1}$ removed. To explore the impact of inherent SBP value and our defined SBP variability on the risk of CV, we consider the following survival submodel,

$$\lambda_{i}(t)=\lambda_{0}(t)\;\text{exp}\;\left\{\gamma^{⊤}w_{i}+\alpha_{1}\left(x_{i}^{⊤}\eta +m_{i}(t)\right)+\alpha_{2}{\left(\int_{0.25}^{t}{\left\{m_{i}^{\prime \prime }(s)\right\}}^{2}ds\right)}^{1/2}\right\},\;t>0.25$$

where $w_{i}=\left(I_{{\text{tmt}}_{i}=1},I_{{\text{tmt}}_{i}=2},{\text{Sex}}_{i},{\text{Smoke}}_{i},{\text{Age}}_{i}\right)$ are baseline covariates, which are determined according to prior knowledge and naive analysis of event times (see Figure 3(b)). Usually in medicine, the initial reduction in SBP is attributed to treatment initiation and dose adjustment as clinical trials aim to achieve good early control of blood pressure (Rothwell *and others*, 2010 *a*). Therefore, the square root of cumulative variability from the 3-month (0.25-year) visit is considered and incorporated in the survival submodel as a predictor.

Table 6 shows the estimation results from the proposed joint model (JM), the standard joint model (No Variability) and the naive proportional hazard (PH) model. Both the proposed and standard joint models are estimated with the algorithm in Section 5 and the standard errors are obtained via bootstrap. The association between CV and the defined measure of SBP variability (*α*_2_), after adjustment for underlying SBP level and baseline covariates, is significant under JM. Specifically, the hazard ratio associated with 1-SD increase in SBP variability (SD for defined SBP variability: 0.535) is 1.14 (95% confidence interval: 1.02–1.27) and 1.20 (95% confidence interval: 1.02–1.40) for underlying SBP level (SD for SBP value: 0.485). The effect of diuretic treatment (*γ*_1_), after adjusting for other baseline predictors, SBP level and variability, still appears to be highly significant in decreasing CV risk, while the effect of *β* blocker (*γ*_2_) is weak. Both joint models lead to similar conclusion on this point. Compared with the estimation results given by PH, the treatment effects (*γ*_1_ and *γ*_2_) are attenuated by incorporating SBP relevant predictors in joint models. It seems that the effects of diuretic and *β* blocker treatments on CV risk can be better captured (explained) through their impact on SBP level and variability together since the treatment effects diminish more in the proposed model than those in the standard joint model. In terms of model performance, the AIC (BIC) values for the proposed and standard joint models are 121 003.6 (121 221.9) and 121 204.3 (121 416.4), respectively.

**Table 6:** Estimation results for MRC data under different models. JM refers to the proposed joint model; No Variability refers to the standard joint model with the restriction $\alpha_{2}=0$ and PH is the proportional hazards model involving baseline covariates only. ${\text{SD}}^{b}$ in both JM and No Variability denote the standard deviation based on 100 bootstrap samples and SD’s in PH are obtained from R function “coxph”.

	JM		No Variability		PH	
Parameters	$\hat{\theta }$	${\text{SD}}^{b}$	$\hat{\theta }$	${\text{SD}}^{b}$	$\hat{\theta }$	SD
**Survival**						
*γ*_1_	–0.4095	0.1389	–0.4425	0.1386	–0.5974	0.1581
*γ*_2_	–0.0967	0.1701	–0.1263	0.1658	–0.2679	0.1386
*γ*_3_	0.7081	0.1228	0.7121	0.1264	0.6827	0.1159
*γ*_4_	0.2219	0.1430	0.2215	0.1405	0.2280	0.1406
*γ*_5_	0.3557	0.2142	0.3584	0.2110	0.3581	0.2092
*α*_1_	0.3693	0.1659	0.3015	0.1372	–	–
*α*_2_	0.2378	0.1035	–	–	–	–
**Longitudinal**						
*η*_1_	–0.0466	0.0106	–0.0471	0.0102	–	–
*η*_2_	0.0330	0.0146	0.0328	0.0147	–	–
*η*_3_	0.0928	0.0208	0.0927	0.0204	–	–
*ξ*	0.0789	0.0049	0.0796	0.0047	–	–
*D*_22_	0.2189	0.0069	0.2219	0.0068	–	–
*D*_33_	0.2161	0.0082	0.2206	0.0080	–	–
*D*_44_	0.4064	0.0175	0.4090	0.0178	–	–
*D*_55_	0.5362	0.0221	0.5395	0.0218	–	–
*D*_66_	0.3037	0.0128	0.3036	0.0125	–	–
$\sigma^{2}$	0.1798	0.0016	0.1798	0.0016	–	–

**Fig. 4:**
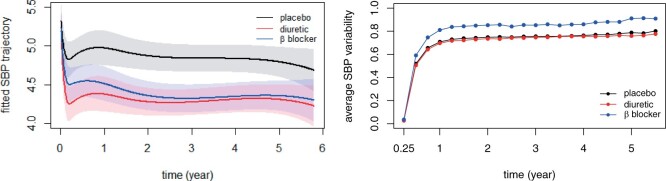
Fitted SBP trajectories and group average SBP variability under JM. Left: fitted SBP mean trends in different treatment groups $\sum_{k=1}^{q}\left({\hat{\beta }}_{k}+I_{\left\{{\text{tmt}}_{i}=1\right\}}{\hat{\beta }}_{k}^{\left(1\right)}+I_{\left\{{\text{tmt}}_{i}=2\right\}}{\hat{\beta }}_{k}^{\left(2\right)}\right)B_{k}(t)$ and corresponding 95% confidence intervals. Right: group average of estimated SBP variability ${\left(\int_{0.25}^{t}{\left\{{\hat{m}}_{i}^{\prime \prime }(s)\right\}}^{2}ds\right)}^{1/2},i=1,\ldots ,m$ by different treatments.

Figure 4 shows the fitted SBP trajectory and variability in each treatment group. Both diuretic and *β* blocker treatments significantly reduce SBP level compared with that in the placebo group. Although the diuretic group exhibits a more immediate drop in SBP value after entry, both active treatment groups reach similar SBP level after two years. However, the square root of cumulative SBP variability appears to be larger in the *β* blocker group than the other two groups, which concurs with existing findings in medicine that *β* blockers tend to induce excessive intraindividual SBP variability over time (Carr *and others*, 2012; Rothwell *and others*, 2010 *a*).

## 8 Discussion

This article focuses on how to characterize the biological variability or instability of underlying biomarker trajectories in joint modeling of longitudinal and time-to-event data. Borrowing ideas from the roughness penalty in smoothing splines and penalized splines, we propose a similar quantity to evaluate the fluctuation of subject-specific longitudinal trajectories which are modeled by mixed-effects regression splines. To support the use of spline bases in modeling longitudinal data and further our proposed variability measure, longitudinal observations should present nonlinear profiles and observation times should not be very sparse. To ensure the $\sqrt{m}$ convergence rate of MLEs, it is required that $\min(n_{i})$ grows at a rate greater than $m^{1/4}$. Otherwise, approximation errors resulting from calculating expectations in the EM algorithm will dominate the estimator’s asymptotic behavior.

The performance of the proposed variability measure depends heavily on the number and locations of knots. For example, trajectories in the right-hand graph of Figure 1 tend to flatten out after time 1 due to the fact that no interior knots are placed in the later period (knot locations in this case are 0.25 and 1.5), whereas similar phenomena do not occur in Figure 2 as the interior knots, in the toy example, are evenly spaced. Increasing the number of knots may alleviate the sensitivity to the locations of knots at the price of increasing model complexity. Although standard methods, e.g., AIC and BIC, provide a way to choose the knot sequence, further exploration and more attention should be given to this issue.

In some cases, it might be more reasonable to use the average variability or the variability over a recent period as a predictor. More generally, we can consider a weighted variability ${\left(\int_{t_{0}}^{t}\omega (t-s){\left\{m_{i}^{\prime \prime }(s)\right\}}^{2}ds\right)}^{1/2}$ where the weight function can be specified within a parametric family with unknown parameters or be modeled in a nonparametric way. The time window over which the biomarker variability is more relevant in predicting survival events can be implied from the estimate of ω(t−s). Since $\int_{t_{0}}^{t}\omega (t-s){\left\{m_{i}^{\prime \prime }(s)\right\}}^{2}ds$ is still a quadratic form of random effects, the methodology developed in this article can be straightforwardly applied to the weighted form.

## 9 Supplementary Material

R code for the simulations is available at https://github.com/cywang0315/R_simulations. Supplementary material is available at http://biostatistics.oxfordjournals.org.

## Supplementary Material

kxad009_Supplementary_Data
