# Supplementary material for: Modeling biomarker variability in joint analysis of longitudinal and time-to-event data
Source: Biostatistics. 2023 May 25;25(2):577–96. doi: 10.1093/biostatistics/kxad009 (PMC11017116; doi:10.1093/biostatistics/kxad009)
Supplement: kxad009_Supplementary_Data [file kxad009_supplementary_data.pdf]

Biostatistics (2023), **0**, 0, *pp.* 1–**??**
doi:10.1093/biostatistics/output

Supplementary materials for "Modelling biomarker variability in joint analysis of longitudinal and time-to-event data"

Chunyu Wang, Jiaming Shen, Christiana Charalambous, and Jianxin Pan*

Department of Mathematics, The University of Manchester, Manchester M13 9PL, U.K

jianxin.pan@manchester.ac.uk

A PROOF

A.1 Conditions

The following conditions are required for Theorem 1 and Theorem 2.

(C.1) Let N_i denote the random variable behind n_i, $i = 1, ..., m$. $P(N_i > q|X_i, Z_i, W_i, T_i) > 0$ with probability one.

(C.2) $P(T_i \geqslant t_0) = 1$.

(C.3) Let $\tilde{X}_i = (X_i, Z_i)$, a design matrix which combines X_i and Z_i by columns. Both $P(\tilde{X}_i^{\top}\tilde{X}_i$ is full rank$)$ and $P(B_i^{\top}B_i$ is full rank$)$ are positive. Moreover, if there exist a constant vector $\boldsymbol{C}_0$ and a constant $\tilde{C}_0$ such that with a positive probability, $W_i^{\top}\boldsymbol{C}_0 = \tilde{C}_0$, then $\boldsymbol{C}_0 = \mathbf{0}$ and $\tilde{C}_0 = 0$.

(C.4) The true parameter $\boldsymbol{\theta}^* = (\gamma^{*\top}, \alpha_1^*, \alpha_2^*, \beta^{*\top}, \eta^{*\top}, \xi^{*\top}, \sigma^{2*}, \text{Vec}(D^*)^{\top})^{\top}$ satisfies $\|\boldsymbol{\theta}^*\|_2 \leqslant M_0$, $\sigma^{2*} > M_0^{-1}$ and $\min_{\|e\|_2=1} e^{\top}D^*e > M_0^{-1}$ for a known positive constant M_0.

*To whom correspondence should be addressed.

© The Author 2023. Published by Oxford University Press. All rights reserved. For permissions, please e-mail: journals.permissions@oup.com

(C.5) The eigenvalues of the matrix collection $\{K(t_0, t), t \in (t_0, \tau]\}$ can be bounded by some finite positive constant $\lambda_{\max}^K$. That is, $\max_{\|e\|_2=1} e^\top K(t_0, t)e \leqslant \lambda_{\max}^K$ for any $t \in (t_0, \tau]$.

(C.6) The true baseline hazard function $\lambda_0^*(t)$ is bounded and positive in $[t_0, \tau]$.

Conditions (C.1) and the first half of (C.3) are imposed to ensure the identifiability of the specified longitudinal submodel. The second half of (C.3) is exactly the linear independence of $[1, W_i]$ with positive probability, which is required to identify the regression coefficients η in our survival submodel in the presence of an unspecified baseline hazard function. For a more general survival submodel, for example, W_i is a time-dependent covariate vector or the linear structure of random effects in the survival submodel is not exactly the same as that in the longitudinal submodel, additional assumptions are required to ensure the identifiablity of parameters in the survival model. See Remark 3.1 in Zeng and Cai (2005) for a related discussion. Condition (C.2) is imposed to accommodate our proposed variability measure which is an integral from t_0 to the current time t. If t_0 is zero, this condition becomes trivial. The case that t_0 is larger than zero is natural if the data comes from a clinical trial where the early stage is manipulated to achieve good control of biomarkers and the event of interest is relatively rare. Condition (C.4) indicates the true value of $\boldsymbol{\theta}$ lies in a compact set, which is a standard regular condition for MLEs. Condition (C.6), along with (C.4), implies $P(T_i = \tau) > 0$, which, combined with (C.5), is essential in proving $\hat{\Lambda}_0(\tau)$ is bounded (see Section A.2, the proof of (ii)).

A.2 Proof of Theorem 1

The proof of Theorem 1 can be established by verifying the following arguments:

(i) The maximum likelihood estimate $(\hat{\boldsymbol{\theta}}, \hat{\Lambda}_0)$ exists.

(ii) With probability one, $\hat{\Lambda}_0(\tau)$ is bounded as m goes to infinity.

(iii) If (ii) is true, then there exists a subsequence of $\hat{\Lambda}_0$ which weakly converges to some right-continuous monotone function $\Lambda_0^{\#}$. Further, we can choose a sub-subsequence of $\hat{\boldsymbol{\theta}}$ which converges to

some $\boldsymbol{\theta}^{\#}$. The third step is to show $\Lambda_0^{\#} = \Lambda_0^*$ and $\boldsymbol{\theta}^{\#} = \boldsymbol{\theta}^*$.

(i) Since $\exp(-x) \leqslant x^{-2}$ for any $x > 0$, we have

$$\Lambda_0\{T_i\} \exp\left(-\int_0^{T_i} \exp\left[\gamma^\top w_i + \alpha_1\{x_i^\top \eta + \tilde{b}_i^\top B(t)\} + \alpha_2 \left\{\tilde{b}_i^\top K(t_0, t)\tilde{b}_i\right\}^{1/2}\right] d\Lambda_0(t)\right)$$
$$\leqslant \frac{1}{\Lambda_0\{T_i\}} \exp\left(-2\left[\gamma^\top w_i + \alpha_1\{x_i^\top \eta + \tilde{b}_i^\top B(T_i)\} + \alpha_2 \left\{\tilde{b}_i^\top K(t_0, T_i)\tilde{b}_i\right\}^{1/2}\right]\right);$$

which leads to

$$l_m(\boldsymbol{\theta}, \Lambda_0) \leqslant$$
$$\sum_{i=1}^m \log\left\{\int \Lambda_0\{T_i\}^{-\delta_i} \exp\left(-\delta_i\left[\gamma^\top w_i + \alpha_1\{x_i^\top \eta + \tilde{b}_i^\top B(T_i)\} + \alpha_2 \left\{\tilde{b}_i^\top K(t_0, T_i)\tilde{b}_i\right\}^{1/2}\right]\right)\right.$$
$$\left. \times \left(\sigma^2\right)^{-n_i/2} \exp\left(-\frac{\left\|Y_i - X_i\eta - \mathrm{B}_i\tilde{b}_i - Z_i\xi\right\|_2^2}{2\sigma^2}\right) |D|^{-1/2} \exp\left(-\frac{b_i^\top D^{-1} b_i}{2}\right) db_i\right\}$$
$$+ C.$$

Therefore, the jump size of $\Lambda_0(\cdot)$ must be finite. Otherwise if $\Lambda_0\{T_i\} \to \infty$ at some uncensored event time T_i, then $l_m(\boldsymbol{\theta}, \Lambda_0) \to -\infty$. On the other hand, $\boldsymbol{\theta}$ belongs to a compact set Θ. Then the maximum likelihood estimate $(\hat{\boldsymbol{\theta}}, \hat{\Lambda}_0)$ exists.

(ii) Define $\hat{\zeta} = \log \hat{\Lambda}_0(\tau)$ and rewrite $\hat{\Lambda}_0(t) = \exp(\hat{\zeta})\tilde{\Lambda}_0(t)$ where $\tilde{\Lambda}_0(t)$ is a rescaled version of $\hat{\Lambda}_0(t)$ with $\tilde{\Lambda}_0(\tau) = 1$. To prove (ii), it is sufficient to show $\hat{\zeta}$ is bounded.

Simple algebraic operations lead to the following expression for $m^{-1} l_m(\hat{\boldsymbol{\theta}}, \Lambda_0)$:

$$-\frac{N}{2m}\log\hat{\sigma}^2 - \frac{1}{2}\log|\hat{D}| - \frac{1}{2}\hat{\beta}^\top\hat{D}^{-1}\hat{\beta} - \frac{1}{m}\sum_{i=1}^m \frac{\|Y_i - X_i\hat{\eta} - Z_i\hat{\xi}\|_2^2}{2\hat{\sigma}^2} + \frac{1}{2m}\sum_{i=1}^m \mu_i^\top V_i \mu_i$$
$$+ \frac{1}{m}\sum_{i=1}^m \log|V_i|^{-1/2} + \frac{1}{m}\sum_{i=1}^m \delta_i\left(\hat{\gamma}^\top w_i + \hat{\alpha}_1 x_i^\top\hat{\eta}\right) + \frac{1}{m}\sum_{i=1}^m \delta_i \log\Lambda_0\{T_i\}$$
$$+ \frac{1}{m}\sum_{i=1}^m \log\int_{b_i^*} \exp\left[-\frac{b_i^{*\top} b_i^*}{2} + \delta_i\alpha_2\left\{\left(\mu_i + V_i^{-1/2} b_i^*\right)^\top K(t_0, T_i)\left(\mu_i + V_i^{-1/2} b_i^*\right)\right\}^{1/2}\right]$$
$$\times \exp\left(-\int_0^{T_i} \exp\left[\hat{\gamma}^\top w_i + \hat{\alpha}_1\left\{x_i^\top\hat{\eta} + B^\top(t)\left(\mu_i + V_i^{-1/2} b_i^*\right)\right\}\right.\right.$$
$$\left.\left. + \hat{\alpha}_2\left\{\left(\mu_i + V_i^{-1/2} b_i^*\right)^\top K(t_0, t)\left(\mu_i + V_i^{-1/2} b_i^*\right)\right\}^{1/2}\right] d\Lambda_0(t)\right) db_i^*$$
$$+ C,$$

where $V_i = \hat{D}^{-1} + \mathrm{B}_i^\top \mathrm{B}_i / \hat{\sigma}^2$ and $\mu_i = V_i^{-1}\left[\mathrm{B}_i^\top (Y_i - X_i\hat{\eta} - Z_i\hat{\xi})/\sigma^2 + \hat{D}^{-1}\hat{\beta} + \delta_i\hat{\alpha}_1 B(T_i)\right]$.

Let

$$R(t, b_i^*, \hat{\boldsymbol{\theta}}) = \left[\left(\mu_i + V_i^{-1/2} b_i^*\right)^\top K(t_0, t)\left(\mu_i + V_i^{-1/2} b_i^*\right)\right]^{1/2},$$

and

$$Q_{1i}(t, \mathbf{b}_i^*, \hat{\boldsymbol{\theta}}) = w_i^\top \hat{\gamma} + \hat{\alpha}_1 x_i^\top \hat{\eta} + \hat{\alpha}_1 B^\top(t)\left(\mu_i + V_i^{-1/2}\mathbf{b}_i^*\right) + \hat{\alpha}_2 R(t, \mathbf{b}_i^*, \boldsymbol{\theta}).$$

Since $0 \leqslant m^{-1} l_m\left(\hat{\boldsymbol{\theta}}, \exp(\hat{\zeta})\tilde{\Lambda}_0\right) - n^{-1} l_m(\hat{\boldsymbol{\theta}}, \tilde{\Lambda}_0)$, it follows that

$$\begin{aligned} 0 \leqslant & \frac{1}{n}\sum_{i=1}^m \delta_i \hat{\zeta} \\ & + \frac{1}{m}\sum_{i=1}^m \log\left(\int_{\mathbf{b}_i^*} \exp\left[-\frac{1}{2}\mathbf{b}_i^{*\top}\mathbf{b}_i^* + \delta_i \hat{\alpha}_2 R(T_i, \mathbf{b}_i^*, \hat{\boldsymbol{\theta}})\right.\right. \\ & \left.\left. \qquad - \exp(\hat{\zeta})\int_0^{T_i} \exp\{Q_{1i}(t, \mathbf{b}_i^*, \hat{\boldsymbol{\theta}})\} d\widetilde{\Lambda}(t)\right] d\mathbf{b}_i^*\right) \\ & - \frac{1}{m}\sum_{i=1}^m \log\left(\int_{\mathbf{b}_i^*} \exp\left[-\frac{1}{2}\mathbf{b}_i^{*\top}\mathbf{b}_i^* + \delta_i \hat{\alpha}_2 R(T_i, \mathbf{b}_i^*, \hat{\boldsymbol{\theta}})\right.\right. \\ & \left.\left. \qquad - \int_0^{T_i} \exp\{Q_{1i}(t, \mathbf{b}_i^*, \hat{\boldsymbol{\theta}})\} d\widetilde{\Lambda}(t)\right] d\mathbf{b}_i^*\right) \end{aligned} \tag{A.1}$$

According to condition (C.5), we have

$$\begin{aligned} R(t, \mathbf{b}_i^*, \hat{\boldsymbol{\theta}}) \leqslant & (\lambda_{\max}^K)^{1/2}\left\|\mu_i + V_i^{-1/2}\mathbf{b}_i^*\right\|_2 \\ \leqslant & (\lambda_{\max}^K)^{1/2}\left(\|\mu_i\|_2 + \left\|V_i^{-1/2}\mathbf{b}_i^*\right\|_2\right) \\ \leqslant & C_1 + C_2\|Y_i\|_2 + C_3\|\mathbf{b}_i^*\|_2, \end{aligned} \tag{A.2}$$

and

$$|Q_{1i}(t, \mathbf{b}_i^*, \hat{\boldsymbol{\theta}})| \leqslant C_4 \parallel \mathbf{b}_i^* \parallel_2 + C_5 \parallel Y_i \parallel_2 + C_6, \tag{A.3}$$

where $C_j, j = 1, .., 6$ are some positive constants. Let $\mathrm{E}_\Phi(\cdot)$ denote the expectation with respect to standard multivariate normal distribution, due to the concavity of logarithm function and inequalities

(A.2)&(A.3),

$$
\begin{aligned}
&\log\int_{\mathbf{b}_i^*}\exp\left[\frac{-\mathbf{b}_i^{*\top}\mathbf{b}_i^*}{2}+\delta_i\hat{\alpha}_2R(T_i,\mathbf{b}_i^*,\hat{\boldsymbol{\theta}})-\int_0^{T_i}\exp\{Q_{1i}(t,\mathbf{b}_i^*,\hat{\boldsymbol{\theta}})\}d\widetilde{\Lambda}(t)\right]d\mathbf{b}_i^*\\
=&(2\pi)^{q/2}\log\mathrm{E}_\Phi\left(\exp\left[\delta_i\hat{\alpha}_2R(T_i,\mathbf{b}_i^*,\hat{\boldsymbol{\theta}})-\int_0^{T_i}\exp\{Q_{1i}(t,\mathbf{b}_i^*,\hat{\boldsymbol{\theta}})\}d\tilde{\Lambda}(t)\right]\right)\\
\geqslant&(2\pi)^{q/2}\mathrm{E}_\Phi\left[\delta_i\hat{\alpha}_2R(T_i,\mathbf{b}_i^*,\hat{\boldsymbol{\theta}})-\int_0^{T_i}\exp\{Q_{1i}(t,\mathbf{b}_i^*,\hat{\boldsymbol{\theta}})\}d\tilde{\Lambda}(t)\right]\\
\geqslant&(2\pi)^{q/2}\left[-|\hat{\alpha}_2|(\lambda_{\max}^K)^{1/2}\mathrm{E}_\Phi\left(\|\mu_i+V_i^{-1/2}\mathbf{b}_i^*\|_2\right)+\mathrm{E}_\Phi\left\{-\exp\left(C_4\left\|\mathbf{b}^*\right\|_2+C_5\left\|\mathbf{Y}_i\right\|_2+C_6\right)\right\}\right]\\
=&-\exp\left(C_5\left\|\mathbf{Y}_i\right\|_2+C_7\right)-C_8\left\|\mathbf{Y}_i\right\|_2-C_9
\end{aligned}
$$

where C_7, C_8 and C_9 are another three positive constants. Thus, the third term in (A.1) satisfies

$$
\begin{aligned}
&-\frac{1}{m}\sum_{i=1}^m\log\left(\int_{\mathbf{b}_i^*}\exp\left[-\frac{1}{2}\mathbf{b}_i^{*\top}\mathbf{b}_i^*+\delta_i\hat{\alpha}_2R(T_i,\mathbf{b}_i^*,\hat{\boldsymbol{\theta}})-\int_0^{T_i}\exp\{Q_{1i}(t,\mathbf{b}_i^*,\hat{\boldsymbol{\theta}})\}d\widetilde{\Lambda}(t)\right]d\mathbf{b}_i^*\right)\\
&\leqslant C_9+\frac{1}{m}\sum_{i=1}^m\left\{\exp\left(C_5\left\|\mathbf{Y}_i\right\|_2+C_7\right)+C_8\left\|\mathbf{Y}_i\right\|_2\right\},
\end{aligned}
$$

which, by the strong law of large numbers, can be bounded by some constant C_{10}. Then (A.1) becomes

$$\begin{aligned}
0 \leqslant & \frac{1}{m}\sum_{i=1}^{m}\delta_i\hat{\zeta} \\
& + \frac{1}{m}\sum_{i=1}^{m}\log\left(\int_{\mathbf{b}_i^*}\exp\left[-\frac{1}{2}\mathbf{b}_i^{*\top}\mathbf{b}_i^* + \delta_i\hat{\alpha}_2 R(T_i,\mathbf{b}_i^*,\hat{\boldsymbol{\theta}})\right.\right. \\
& \left.\left. \qquad - \exp(\hat{\xi})\int_0^{T_i}\exp\{Q_{1i}(t,\mathbf{b}_i^*,\hat{\boldsymbol{\theta}})\}d\widetilde{\Lambda}(t)\right]d\mathbf{b}_i^*\right) + C_{10} \\
\leqslant & \frac{1}{m}\sum_{i=1}^{m}\delta_i\hat{\zeta} \\
& + \frac{1}{m}\sum_{i=1}^{m}I(T_i=\tau)\log\left(\int_{\mathbf{b}_i^*}\exp\left[-\frac{1}{2}\mathbf{b}_i^{*\top}\mathbf{b}_i^* + \delta_i\hat{\alpha}_2 R(T_i,\mathbf{b}_i^*,\hat{\boldsymbol{\theta}})\right.\right. \\
& \left.\left. \qquad - \exp(\hat{\xi})\int_0^{\tau}\exp\{Q_{1i}(t,\mathbf{b}_i^*,\hat{\boldsymbol{\theta}})\}d\widetilde{\Lambda}(t)\right]d\mathbf{b}_i^*\right) \\
& + \frac{1}{m}\sum_{i=1}^{m}I(T_i\neq\tau)\log\left[\int_{\mathbf{b}_i^*}\exp\left\{-\frac{1}{2}\mathbf{b}_i^{*\top}\mathbf{b}_i^* + \delta_i\hat{\alpha}_2 R(T_i,\mathbf{b}_i^*,\hat{\boldsymbol{\theta}})\right\}d\mathbf{b}_i^*\right] + C_{10} \\
\leqslant & \frac{1}{m}\sum_{i=1}^{m}\delta_i\hat{\zeta} \\
& + \frac{1}{m}\sum_{i=1}^{m}I(T_i=\tau)\log\left(\int_{\mathbf{b}_i^*}\exp\left[-\frac{1}{2}\mathbf{b}_i^{*\top}\mathbf{b}_i^* + \delta_i\hat{\alpha}_2 R(T_i,\mathbf{b}_i^*,\hat{\boldsymbol{\theta}})\right.\right. \\
& \left.\left. \qquad - \exp(\hat{\xi})\int_0^{\tau}\exp\{Q_{1i}(t,\mathbf{b}_i^*,\hat{\boldsymbol{\theta}})\}d\widetilde{\Lambda}(t)\right]d\mathbf{b}_i^*\right) + C_{11},
\end{aligned} \tag{A.4}$$

where C_{11} is a constant.

Additionally, using the inequality that $\exp(-x) \leqslant (1+x/\Gamma)^{-\Gamma}$ for any $\Gamma > 0$ and $x > 0$, we have

$$\begin{aligned}
& \exp\left[\frac{-\mathbf{b}_i^{*\top}\mathbf{b}_i^*}{2} + \delta_i\hat{\alpha}_2 R(T_i,\mathbf{b}_i^*,\hat{\boldsymbol{\theta}}) - \exp(\hat{\zeta})\int_0^{\tau}\exp\{Q_{1i}(t,\mathbf{b}_i^*,\hat{\boldsymbol{\theta}})\}d\widetilde{\Lambda}(t)\right] \\
\leqslant & \exp\left\{\frac{-\mathbf{b}_i^{*\top}\mathbf{b}_i^*}{2} + \delta_i\hat{\alpha}_2 R(T_i,\mathbf{b}_i^*,\hat{\boldsymbol{\theta}})\right\}\left[1+\exp(\hat{\zeta})\int_0^{\tau}\exp\{Q_{1i}(t,\mathbf{b}_i^*,\hat{\boldsymbol{\theta}})\}d\widetilde{\Lambda}(t)/\Gamma\right]^{-\Gamma} \\
\leqslant & \Gamma^{\Gamma}\exp\left\{\frac{-\mathbf{b}_i^{*\top}\mathbf{b}_i^*}{2} + \delta_i\hat{\alpha}_2 R(T_i,\mathbf{b}_i^*,\hat{\boldsymbol{\theta}}) - \Gamma\hat{\zeta}\right\}\left(\int_0^{\tau}\exp\{Q_{1i}(t,\mathbf{b}_i^*,\hat{\boldsymbol{\theta}})\}d\widetilde{\Lambda}(t)\right)^{-\Gamma}
\end{aligned} \tag{A.5}$$

Substituting (A.5) into (A.4) leads to the following expression

$$
\begin{aligned}
0 \leqslant & \frac{1}{m}\sum_{i=1}^{m}\delta_i\hat{\zeta}+C_{11} \\
& + \frac{1}{m}\sum_{i=1}^{m} I(T_i=\tau)\log\Bigg(\Gamma^{\Gamma}\exp(-\Gamma\hat{\zeta})\int_{\mathbf{b}_i^*}\exp\left\{\frac{-\mathbf{b}_i^{*\top}\mathbf{b}_i^*}{2}+\delta_i\hat{\alpha}_2 R(T_i,\mathbf{b}_i^*,\hat{\boldsymbol{\theta}})\right\} \\
& \qquad \times\left[\int_0^{\tau}\exp\{Q_{1i}(t,\mathbf{b}_i^*,\hat{\boldsymbol{\theta}})\}d\widetilde{\Lambda}(t)\right]^{-\Gamma} d\mathbf{b}_i^*\Bigg) \\
\leqslant & \frac{1}{m}\sum_{i=1}^{m}\delta_i\hat{\zeta}+C_{11} \\
& + \frac{1}{m}\sum_{i=1}^{m} I(T_i=\tau)\log\Bigg[\Gamma^{\Gamma}\exp(-\Gamma\hat{\zeta}) \\
& \qquad \times\int_{\mathbf{b}_i^*}\exp\Bigg\{\frac{-\mathbf{b}_i^{*\top}\mathbf{b}_i^*}{2}+|\hat{\alpha}_2|\left(C_1+C_2\|Y_i\|_2+C_3\|\mathbf{b}_i^*\|_2\right) \\
& \qquad +\Gamma\left(C_4\|\mathbf{b}_i^*\|_2+C_5\|Y_i\|_2+C_6\right)\Bigg\}d\mathbf{b}_i^*\Bigg] \\
\leqslant & C_{11}+\left(\frac{1}{m}\sum_{i=1}^{m}\delta_i\right)\hat{\zeta}-\left(\frac{\Gamma}{m}\sum_{i=1}^{m}I(T_i=\tau)\right)\hat{\zeta}+C_{12}(\Gamma),
\end{aligned}
\tag{A.6}
$$

where $C_{12}(\Gamma)$ is a deterministic function of Γ. By the strong law of large numbers, $\sum_{i=1}^{m} I(T_i=\tau)/m \overset{a.s.}{\to} \mathrm{P}(T=\tau)>0$. Thus we can choose Γ large enough such that

$$
\frac{\Gamma}{m}\sum_{i=1}^{m}I(T_i=\tau)\geqslant\frac{2}{m}\sum_{i=1}^{m}\delta_i.
$$

In this way (A.6) becomes

$$
0\leqslant C_{11}-\left(\frac{1}{m}\sum_{i=1}^{m}\delta_i\right)\hat{\zeta}+C_{12}(\Gamma),
$$

from which we obtain an upper bound for $\hat{\Lambda}(\tau)$, i.e., $\hat{\Lambda}(\tau)\leqslant\exp(m(C_{11}+C_{12}(\Gamma))/\sum_{i=1}^{m}\delta_i)$. Thus, we have proved that with probability 1, $\hat{\Lambda}(\tau)$ is bounded for any sample size m.

(iii) Let O_i denote the collection of the i-th observed data $(Y_i,x_i,n_i,\{t_{ij}\}_{j=1}^{n_i},T_i,\delta_i,w_i)$ and use O with subscript i removed to denote the corresponding random variables. Differentiating $l_m(\boldsymbol{\theta},\Lambda_0)$ with respect to the jump size $\Lambda_0\{T_i\}$, we obtain that $\hat{\Lambda}_0\{T_i\}$ satisfies the equation

$$
\hat{\Lambda}_0\{T_i\}=\frac{\delta_i}{\sum_{j=1}^{m}I(T_j\geqslant T_i)\mathrm{E}[r(b_j,T_i,\hat{\boldsymbol{\theta}})|T_j,\delta_j,Y_j;\hat{\boldsymbol{\theta}},\hat{\Lambda}_0]}. \tag{A.7}
$$

To express concisely, we define $G(b_i, O_i; \boldsymbol{\theta}, \Lambda_0)$ as

$$(2\pi\sigma^2)^{-n_i/2} \exp\left\{-\frac{\left\|Y_i - X_i\eta - \mathrm{B}_i\tilde{b}_i - Z_i\xi\right\|^2}{2\sigma^2}\right\}(2\pi)^{-q/2}|D|^{-1/2}\exp\left\{-\frac{b_i^{\mathrm{T}}D^{-1}b_i}{2}\right\}$$
$$\times \exp\left\{\delta_i\left[\gamma^\top w_i + \alpha_1(x_i^\top\eta + \tilde{b}_i^\top B(T_i)) + \alpha_2\left(\tilde{b}_i^\top K(t_0, T_i)\tilde{b}_i\right)^{1/2}\right]\right.$$
$$\left. - \int_0^{T_i} \exp\left(\gamma^\top w_i + \alpha_1(x_i^\top\eta + \tilde{b}_i^\top B(t)) + \alpha_2\left(\tilde{b}_i^\top K(t_0, t)\tilde{b}_i\right)^{1/2}\right) d\Lambda_0(t)\right\}$$

and further define

$$Q(t, O_i; \boldsymbol{\theta}, \Lambda_0) = \frac{\int_{b_i} G(b_i, O_i; \theta, \Lambda_0) r(b_i, T_i, \boldsymbol{\theta}) db_i}{\int_{b_i} G(b_i, O_i; \theta, \Lambda_0) db_i}.$$

Additionally, we introduce operator notations $\mathbf{P}_m f = m^{-1}\sum_{i=1}^m f(O_i)$ and $\mathbf{P}f = \int f d\mathbf{P} = E[f(O)]$ where $\mathbf{P}_m$ is exactly the empirical measure from m i.i.d observations and $\mathbf{P}$ is the true probability measure which, in our model, is the abbreviation of $\mathbf{P}_{\theta^*, \Lambda_0^*}$. Under notations defined above, (A.7) can be rewritten as

$$\hat{\Lambda}_0\{T_i\} = \frac{\delta_i}{\sum_{j=1}^m I(T_j \geqslant T_i) Q(T_i, O_j; \hat{\boldsymbol{\theta}}, \hat{\Lambda}_0)} = \frac{\delta_i}{m\mathbf{P}_m\{I(T > t)Q(t, O; \hat{\boldsymbol{\theta}}, \hat{\Lambda}_0)\}|_{t=T_i}},$$

and thus $\hat{\Lambda}_0\{t\}$ satisfies the following equation

$$\hat{\Lambda}_0(t) = \frac{1}{m}\sum_{i=1}^m \frac{I(T_i \leqslant t)\delta_i}{\mathbf{P}_m\{I(T \geqslant s)Q(s, O; \hat{\boldsymbol{\theta}}, \hat{\Lambda}_0)\}|_{s=T_i}} = \mathbf{P}_m\left[\frac{I(T \leqslant t)\delta}{\mathbf{P}_m\Big\{I(T > s)Q(s, O; \hat{\boldsymbol{\theta}}, \hat{\Lambda}_0)\Big\}|_{s=T}}\right].$$

On the other hand, derivations similar to that in Tsiatis (1981) yield the expression for the true baseline hazard function

$$\Lambda_0^*(t) = \mathbf{P}\left[\frac{I(T \leqslant t)\delta}{\mathbf{P}\left\{I(T > s)Q(s, O; \boldsymbol{\theta}^*, \Lambda_0^*)\right\}|_{s=T}}\right].$$

To establish the connection between $\hat{\Lambda}_0\{t\}$ and $\Lambda_0^*(t)$, we construct another function $\bar{\Lambda}_0(t) \in \mathcal{Z}_m$ with jump size given by

$$\bar{\Lambda}_0\{T_i\} = \frac{\delta_i}{m\mathbf{P}_m\{I(T > t)Q(t, O; \boldsymbol{\theta}^*, \Lambda_0^*)\}|_{t=T_i}}.$$

Equivalently,

$$\bar{\Lambda}_0(t) = \mathbf{P}_m\left[\frac{I(T \leqslant t)\delta}{\mathbf{P}_m\{I(T > s)Q(s, O; \boldsymbol{\theta}^*, \Lambda_0^*)\}|_{s=T}}\right].$$

The conclusion is that $\bar{\Lambda}_0(t)$ uniformly converges to $\Lambda_0^*(t)$ in $[0, \tau]$. To prove this, we focus on the difference between $\bar{\Lambda}_0(t)$ and $\Lambda_0^*(t)$:

$$\begin{aligned}
&\sup_{t\in[0,\tau]}\left|\mathbf{P}_m\left[\frac{I(T\leqslant t)\delta}{\mathbf{P}_m\{I(T>s)Q(s,O;\boldsymbol{\theta}^*,\Lambda_0^*)\}\,|_{s=T}}\right]-\mathbf{P}\left[\frac{I(T\leqslant t)\delta}{\mathbf{P}\left\{I(T>s)Q(s,O;\boldsymbol{\theta}^*,\Lambda_0^*)\right\}|_{s=T}}\right]\right|\\
&\leqslant \sup_{t\in[0,\tau]}\left|\frac{1}{m}\sum_{i=1}^{m}I(Z_i\leqslant t)\delta_i\left[\frac{1}{\mathbf{P}_m\{I(T>s)Q(s,O;\boldsymbol{\theta}^*,\Lambda_0^*)\}}-\frac{1}{\mathbf{P}\{I(T>s)Q(s,O;\boldsymbol{\theta}^*,\Lambda_0^*)\}}\right]|_{s=T_i}\right|\\
&\quad+\sup_{t\in[0,\tau]}\left|(\mathbf{P}_m-\mathbf{P})\left[\frac{I(T\leqslant t)\delta}{\mathbf{P}\left\{I(T>s)Q(s,O;\boldsymbol{\theta}^*,\Lambda_0^*)\right\}|_{s=T}}\right]\right|\\
&\leqslant \sup_{t\in[0,\tau]}\left|\left[\frac{1}{\mathbf{P}_m\{I(T>t)Q(t,O;\boldsymbol{\theta}^*,\Lambda_0^*)\}}-\frac{1}{\mathbf{P}\{I(T>t)Q(t,O;\boldsymbol{\theta}^*,\Lambda_0^*)\}}\right]\right|\\
&\quad+\sup_{t\in[0,\tau]}\left|(\mathbf{P}_m-\mathbf{P})\left[\frac{I(T\leqslant t)\delta}{\mathbf{P}\left\{I(T>s)Q(s,O;\boldsymbol{\theta}^*,\Lambda_0^*)\right\}|_{s=T}}\right]\right|.
\end{aligned} \tag{A.8}$$

Since both classes $\{I(T \leqslant t)\delta/\mathbf{P}\left\{I(T > s)Q(s, O; \boldsymbol{\theta}^*, \Lambda_0^*)\right\}|_{s=T} : t \in [0, \tau]\}$ and $\{I(T > t)Q(t, O; \boldsymbol{\theta}^*, \Lambda_0^*) : t \in [0, \tau]\}$ are Glivenko-Cantelli classes, the two terms on the left-hand side of (A.8) converge to zero. Therefore, we arrive at the conclusion that $\bar{\Lambda}_0(t)$ uniformly converges to $\Lambda_0^*(t)$.

Additionally, the definition of $\hat{\Lambda}_0(t)$ gives

$$\hat{\Lambda}_0(t) = \int_0^t \frac{\mathbf{P}_m\left\{I(T \geqslant s)Q\left(s, O; \boldsymbol{\theta}^*, \Lambda_0^*\right)\right\}}{\mathbf{P}_m\{I(T \geqslant s)Q(s, O; \hat{\boldsymbol{\theta}}, \hat{\Lambda}_0)\}} d\bar{\Lambda}_0(s). \tag{A.9}$$

(A.9) implies $\hat{\Lambda}_0(t)$ is absolutely continuous with respect to $\bar{\Lambda}_0(t)$ and the Radon-Nikodym derivative is given by

$$\frac{d\hat{\Lambda}_0(t)}{d\bar{\Lambda}_0(t)} = \frac{\mathbf{P}_m\left\{I(T \geqslant t)Q\left(t, O; \boldsymbol{\theta}^*, \Lambda_0^*\right)\right\}}{\mathbf{P}_m\{I(T \geqslant t)Q(t, O; \hat{\boldsymbol{\theta}}, \hat{\Lambda}_0)\}}.$$

On the other hand, the conclusion of (ii) implies there exists a subsequence of $\hat{\Lambda}_0$ which weakly converges to some right-continuous monotone function $\Lambda_0^{\#}$. Further, we can choose a sub-subsequence of $\hat{\boldsymbol{\theta}}$ which converges to some $\boldsymbol{\theta}^{\#}$. What we need to do next is to show $\Lambda_0^{\#} = \Lambda_0^*$ and $\boldsymbol{\theta}^{\#} = \boldsymbol{\theta}^*$. Our discussions below are restricted to the sub-subsequence satisfying $\hat{\Lambda}_0 \to \Lambda_0^{\#}$ and $\hat{\boldsymbol{\theta}} \to \boldsymbol{\theta}^{\#}$.

Using the bounded convergence theorem, we have that $\mathbf{P}\left\{I(T \geqslant t)Q(t, O; \hat{\boldsymbol{\theta}}, \hat{\Lambda}_0)\right\}$ converges to $\mathbf{P}\left\{I(T \geqslant t)Q(t, O; \boldsymbol{\theta}^{\#}, \Lambda_0^{\#})\right\}$ for each t. Moreover, $\mathbf{P}\left\{I(T \geqslant t)Q(t, O; \hat{\boldsymbol{\theta}}, \hat{\Lambda}_0)\right\}$ is equi-continuous

with respect to t. Then by Arzelà–Ascoli theorem, uniformly in $t \in [0,\tau]$,

$$\mathbf{P}\left\{I(T \geqslant t)Q(t,O;\hat{\boldsymbol{\theta}},\hat{\Lambda}_0)\right\} \to \mathbf{P}\left\{I(T \geqslant t)Q(t,O;\boldsymbol{\theta}^{\#},\Lambda_0^{\#})\right\}.$$

Therefore, uniformly in $t \in [0,\tau]$

$$\frac{\hat{\Lambda}_0\{t\}}{\bar{\Lambda}_0\{t\}} = \frac{\mathbf{P}_m\left\{I(T \geqslant t)Q\left(t,O;\boldsymbol{\theta}^*,\Lambda_0^*\right)\right\}}{\mathbf{P}_m\{I(T \geqslant t)Q(t,O;\hat{\boldsymbol{\theta}},\hat{\Lambda}_0)\}} \to \frac{\mathbf{P}\left\{I(T \geqslant t)Q\left(t,O;\boldsymbol{\theta}^*,\Lambda_0^*\right)\right\}}{\mathbf{P}\{I(T \geqslant t)Q(t,O;\boldsymbol{\theta}^{\#},\Lambda_0^{\#})\}}. \tag{A.10}$$

Applying (A.10) and taking limits on both sides of (A.9) gives

$$\Lambda_0^{\#}(t) = \int_0^t \frac{\mathbf{P}\left\{I(T \geqslant s)Q\left(s,O;\boldsymbol{\theta}^*,\Lambda_0^*\right)\right\}}{\mathbf{P}\{I(T \geqslant s)Q(s,O;\boldsymbol{\theta}^{\#},\Lambda_0^{\#})\}} d\Lambda_0^*(s). \tag{A.11}$$

Since $\Lambda_0^*(t)$ is differentiable with respect to the Lebesgue measure, so is $\Lambda_0^{\#}(t)$ and we denote the derivative of $\Lambda_0^{\#}(t)$ by $\lambda_0^{\#}(t)$. Additionally, (A.10) combined with (A.11) indicates that $\hat{\Lambda}\{t\}/\bar{\Lambda}\{t\}$ uniformly converges to $\lambda_0^{\#}(t)/\lambda_0^*(t)$. Moreover, $\hat{\Lambda}_0(t)$ uniformly converges to $\Lambda_0^{\#}(t)$.

On the other hand,

$$\begin{aligned}0 \leqslant & m^{-1}l_m(\hat{\boldsymbol{\theta}},\hat{\Lambda}_0) - m^{-1}l_m\left(\boldsymbol{\theta}^*,\bar{\Lambda}_0\right) \\ =& \mathbf{P}_m\left[\delta \log \frac{\hat{\Lambda}_0\{T\}}{\bar{\Lambda}_0\{T\}}\right] + \mathbf{P}_m\left[\log \frac{\int G(b,O;\hat{\boldsymbol{\theta}},\hat{\Lambda}_0)db}{\int G\left(b,O;\boldsymbol{\theta}^*,\bar{\Lambda}_0\right)db}\right].\end{aligned} \tag{A.12}$$

Since $\log[\int G(b,O;\hat{\boldsymbol{\theta}},\hat{\Lambda}_0)db/\int G\left(b,O;\boldsymbol{\theta}^*,\bar{\Lambda}_0\right)db]$ belongs to a Glivenko-Canteli class and again using bound converge theorem, we have

$$\mathbf{P}_m\left[\log \frac{\int G(b,O;\hat{\boldsymbol{\theta}},\hat{\Lambda}_0)db}{\int G\left(b,O;\boldsymbol{\theta}^*,\bar{\Lambda}_0\right)db}\right] \to \mathbf{P}\left[\log \frac{\int G(b,O;\boldsymbol{\theta}^{\#},\Lambda_0^{\#})db}{\int G\left(b,O;\boldsymbol{\theta}^*,\Lambda_0^*\right)db}\right].$$

Similarly,

$$\mathbf{P}_m\left[\delta \log \frac{\hat{\Lambda}_0\{T\}}{\bar{\Lambda}_0\{T\}}\right] \to \mathbf{P}\left[\delta \log \frac{\lambda_0^{\#}(T)}{\lambda_0^*(T)}\right]$$

as $\hat{\Lambda}\{t\}/\bar{\Lambda}\{t\} \to \lambda_0^{\#}(t)/\lambda_0(t)$ uniformly in $t \in [0,\tau]$. Thus, taking limits on both side of (A.12) gives

$$\mathbf{P}\left[\log \frac{\lambda_0^{\#}(T)^\delta \int G(b,O;\boldsymbol{\theta}^{\#},\Lambda_0^{\#})db}{\lambda_0^*(T)^\delta \int G\left(b,O;\boldsymbol{\theta}^*,\Lambda_0^*\right)db}\right] \geqslant 0.$$

However, the left-hand side of the inequality above is exactly the negative Kullback–Leibler divergence. Then it follows that, with probability one,

$$\lambda_0^{\#}(T)^\delta \int G(b,O;\boldsymbol{\theta}^{\#},\Lambda_0^{\#})db = \lambda_0^*(T)^\delta \int G\left(b,O;\boldsymbol{\theta}^*,\Lambda_0^*\right)db. \tag{A.13}$$

Under assumptions specified in the beginning, our proposed joint model is identifiable and thus we conclude from (A.13) that $\boldsymbol{\theta}^{\#} = \boldsymbol{\theta}^{*}$ and $\Lambda_0^{\#} = \Lambda_0^{*}$.

A.3 Proof of Theorem 2

Consider the submodel $\mathrm{P}_{\theta_\varepsilon, \Lambda_\varepsilon}$, where $\theta_\varepsilon = \theta^* + \varepsilon \mathbf{h}_1$ and $\Lambda_\varepsilon(t) = \int_0^t (1 + \varepsilon h_2(s)) d\Lambda_0^*(s)$ with $(\mathbf{h}_1, h_2)$ being an element in the set

$$\mathscr{H} = \left\{ (\mathbf{h}_1, h_2) : \|\mathbf{h}_1\| \leqslant 1, \|h_2\|_V \leqslant 1 \right\},$$

where $\|h_2\|_V$ is the total variation of h_2 is $[0, \tau]$. Let $\psi = (\theta, \Lambda_0) \in \Psi = \{(\theta, \Lambda_0) : \|\boldsymbol{\theta} - \boldsymbol{\theta}^*\| + \sup_{t \in [0,\tau]} |\Lambda_0(t) - \Lambda_0^*(t)| \leqslant \delta\}$ for a fixed small constant δ. Define

$$S_m(\psi)(\mathbf{h}_1, h_2) = \mathbf{P}_m \left\{ \dot{l}_\theta(\boldsymbol{\theta}, \Lambda_0)^T \mathbf{h}_1 + \dot{l}_{\Lambda_0}(\boldsymbol{\theta}, \Lambda_0)[h_2] \right\}$$
$$S(\psi)(\mathbf{h}_1, h_2) = \mathbf{P} \left\{ \dot{l}_\theta(\boldsymbol{\theta}, \Lambda_0)^T \mathbf{h}_1 + \dot{l}_{\Lambda_0}(\boldsymbol{\theta}, \Lambda_0)[h_2] \right\},$$

where $\dot{l}_\theta(\theta, \Lambda_0)$ is the derivative of the log-likelihood function from one single subject $l(O; \boldsymbol{\theta}, \Lambda_0)$ with respect to $\boldsymbol{\theta}$, and $\dot{l}_{\Lambda_0}(\theta, \Lambda_0)[h_2]$ is the derivative of $l(O; \boldsymbol{\theta}, \Lambda_\varepsilon)$ at $\varepsilon = 0$. Clearly S_n and S can be viewed as maps from Ψ to $l^\infty(\mathscr{H})$. We denote $(h_1^\sigma, \mathbf{h}_1^b, \mathbf{h}_1^\beta, \mathbf{h}_1^\eta, \mathbf{h}_1^\xi, h_1^{\alpha_1}, h_1^{\alpha_2}, \mathbf{h}_1^\gamma)$ as the corresponding components of $\mathbf{h}_1$ for the parameters $(\sigma, D, \beta, \eta, \xi, \alpha_1, \alpha_2, \gamma)$, then $\dot{l}_\theta(\boldsymbol{\theta}, \Lambda_0)^T \mathbf{h}_1 + \dot{l}_{\Lambda_0}(\boldsymbol{\theta}, \Lambda_0)[h_2]$ has the following specific expression:

$$\mu_1(O; \boldsymbol{\theta}, \Lambda_0)^T \mathbf{h}_1 - \int_0^T \mu_2(t, O; \boldsymbol{\theta}, \Lambda_0)^T \mathbf{h}_1 d\Lambda_0(t) + \delta h_2(T) - \int_0^T \mu_3(t, O; \boldsymbol{\theta}, \Lambda_0) h_2(t) d\Lambda_0(t),$$

where

$$
\begin{aligned}
&\mu_1(O;\boldsymbol{\theta},\Lambda_0)^T\mathbf{h}_1\\
&=\left\{\int_b G(b,O;\boldsymbol{\theta},\Lambda_0)db\right\}^{-1}\\
&\times\int_b G(b,O;\boldsymbol{\theta},\Lambda_0)\Bigg\{\frac{b^T D^{-1}\mathcal{M}_b D^{-1}b}{2}-\operatorname{tr}\left(D^{-1}\mathcal{M}_b\right)\\
&\quad+\frac{\left\|Y-X\eta-\mathrm{B}\,\tilde{b}-Z\xi\right\|^2 h_1^{\sigma}}{\sigma^3}-\frac{Nh_1^{\sigma}}{\sigma}\\
&\quad+\delta\left[\alpha_1 B^{\top}(T)+\alpha_2\left(\tilde{b}^{\top}K(t_0,T)\tilde{b}\right)^{-1/2}\tilde{b}^{\top}K(t_0,T)\right]\mathbf{h}_1^{\beta}+\frac{\left(Y-X\eta-\mathrm{B}\,\tilde{b}-Z\xi\right)^{\top}\mathrm{B}}{\sigma^2}\mathbf{h}_1^{\beta}\\
&\quad+\delta\alpha_1 x^{\top}\mathbf{h}_1^{\eta}+\frac{\left(Y-X\eta-\mathrm{B}\,\tilde{b}-Z\xi\right)^{\top}X}{\sigma^2}\mathbf{h}_1^{\eta}+\frac{\left(Y-X\eta-\mathrm{B}\,\tilde{b}-Z\xi\right)^{\top}Z}{\sigma^2}\mathbf{h}_1^{\xi}\\
&\quad+\delta\left[w^{\top}\mathbf{h}_1^{\gamma}+\left(x^{\top}\eta+B^{\top}(T)\tilde{b}\right)h_1^{\alpha_1}+\left(\tilde{b}^{\top}K(t_0,T)\tilde{b}\right)^{1/2}h_1^{\alpha_2}\right]\Bigg\}db,
\end{aligned}
$$

$$
\begin{aligned}
&\mu_2(t,O;\boldsymbol{\theta},\Lambda_0)^T\mathbf{h}_1\\
&=\left\{\int_b G(b,O;\boldsymbol{\theta},\Lambda_0)db\right\}^{-1}\\
&\times\int_b G(b,O;\boldsymbol{\theta},\Lambda_0)\exp\left\{\gamma^{\top}w+\alpha_1\left(x^{\top}\eta+B^{\top}(t)\tilde{b}\right)+\alpha_2\left(\tilde{b}^{\top}K(t_0,t)\tilde{b}\right)^{1/2}\right\}\\
&\quad\times\delta\Bigg\{w^{\top}\mathbf{h}_1^{\gamma}+\left(x^{\top}\eta+B^{\top}(t)\tilde{b}\right)h_1^{\alpha_1}+\left(\tilde{b}^{\top}K(t_0,t)\tilde{b}\right)^{1/2}h_1^{\alpha_2}+\alpha_1 x^{\top}\mathbf{h}_1^{\eta}\\
&\qquad+\left[\alpha_1 B^{\top}(t)+\alpha_2\left(\tilde{b}^{\top}K(t_0,t)\tilde{b}\right)^{-1/2}\tilde{b}^{\top}K(t_0,t)\right]\mathbf{h}_1^{\beta}\Bigg\}db,
\end{aligned}
$$

and

$$
\begin{aligned}
&\mu_3(t,O;\boldsymbol{\theta},\Lambda_0)\\
&=\left\{\int_b G(b,O;\boldsymbol{\theta},\Lambda_0)db\right\}^{-1}\\
&\times\int_b G(b,O;\boldsymbol{\theta},\Lambda_0)\exp\left\{\gamma^{\top}w+\alpha_1\left(x^{\top}\eta+B^{\top}(t)\tilde{b}\right)+\alpha_2\left(\tilde{b}^{\top}K(t_0,t)\tilde{b}\right)^{1/2}\right\}db.
\end{aligned}
$$

Here, $\mathcal{M}_b$ in $\mu_1(O;\boldsymbol{\theta},\Lambda_0)^T\mathbf{h}_1$ is a symmetric matrix satisfying $\mathrm{Vec}(\mathcal{M}_b)=\mathbf{h}_1^b$.

Let $\nabla_\theta\mu_j$ and $\nabla_{\Lambda_0}\mu_j[\Delta\Lambda_0]$ denote the derivative of μ_j with respect to $\boldsymbol{\theta}$ and Λ_0 along the path $\Lambda_0+\varepsilon\Delta\Lambda_0$ for $j=1,2,3$. Moreover, it's easy to verify that $\nabla_{\Lambda_0}\mu_j[\Delta\Lambda_0]=\int_0^t\mu_{j+3}(s,\mathbf{O};\boldsymbol{\theta},\Lambda_0)d\Delta\Lambda_0(s)$.

Then by mean value theorem, we have

$$
\begin{aligned}
& \dot{l}_\theta(\boldsymbol{\theta}, \Lambda_0)^T \mathbf{h}_1 + \dot{l}_{\Lambda_0}(\boldsymbol{\theta}, \Lambda_0)[h_2] - \dot{l}_\theta(\boldsymbol{\theta}^*, \Lambda_0^*)^T \mathbf{h}_1 - \dot{l}_{\Lambda_0}(\boldsymbol{\theta}^*, \Lambda_0^*)[h_2] \\
&= (\boldsymbol{\theta} - \boldsymbol{\theta}^*)^\top \nabla_\theta \mu_1(\mathbf{O}; \tilde{\boldsymbol{\theta}}, \tilde{\Lambda}_0)\mathbf{h}_1 + \int_0^T \mu_4(t, \mathbf{O}; \tilde{\boldsymbol{\theta}}, \tilde{\Lambda}_0)^\top \mathbf{h}_1 d(\Lambda_0 - \Lambda_0^*)(t) \\
&\quad - (\boldsymbol{\theta} - \boldsymbol{\theta}^*)^\top \int_0^T \nabla_\theta \mu_2\left(t, \mathbf{O}; \tilde{\boldsymbol{\theta}}, \tilde{\Lambda}_0\right) d\Lambda_0(t)\mathbf{h}_1 \\
&\quad - \mathbf{h}_1^\top \int_0^T \left(\int_0^t \mu_5(s, \mathbf{O}; \tilde{\boldsymbol{\theta}}, \tilde{\Lambda}_0) d(\Lambda_0 - \Lambda_0^*)(s)\right) d\Lambda_0(t) \\
&\quad - \int_0^T \mu_2(t, \mathbf{O}; \boldsymbol{\theta}^*, \Lambda_0^*)^\top \mathbf{h}_1 d(\Lambda_0 - \Lambda_0^*)(t) \\
&\quad - (\boldsymbol{\theta} - \boldsymbol{\theta}^*)^\top \int_0^T \nabla_\theta \mu_3\left(t, \mathbf{O}; \tilde{\boldsymbol{\theta}}, \tilde{\Lambda}_0\right) h_2(t) d\Lambda_0(t) \\
&\quad - \int_0^T \left(\int_0^t \mu_6(s, \mathbf{O}; \tilde{\boldsymbol{\theta}}, \tilde{\Lambda}_0) d(\Lambda_0 - \Lambda_0^*)(s)\right) h_2(t) d\Lambda_0(t) \\
&\quad - \int_0^T \mu_3(t, \mathbf{O}; \boldsymbol{\theta}^*, \Lambda_0^*) h_2(t) d(\Lambda_0 - \Lambda_0^*)(t) \\
&= (\boldsymbol{\theta} - \boldsymbol{\theta}^*)^\top \left\{\nabla_\theta \mu_1(\mathbf{O}; \tilde{\boldsymbol{\theta}}, \tilde{\Lambda}_0) - \int_0^T \nabla_\theta \mu_2\left(t, \mathbf{O}; \tilde{\boldsymbol{\theta}}, \tilde{\Lambda}_0\right) d\Lambda_0(t)\right\} \mathbf{h}_1 \\
&\quad + \mathbf{h}_1^\top \int_0^\tau I(t \leqslant T)\left\{\mu_4(t, \mathbf{O}; \tilde{\boldsymbol{\theta}}, \tilde{\Lambda}_0) - \mu_2(t, \mathbf{O}; \boldsymbol{\theta}^*, \Lambda_0^*) - \mu_5(t, \mathbf{O}; \tilde{\boldsymbol{\theta}}, \tilde{\Lambda}_0)\int_t^T d\Lambda_0(s)\right\} d(\Lambda_0 - \Lambda_0^*)(t) \\
&\quad - (\boldsymbol{\theta} - \boldsymbol{\theta}^*)^\top \int_0^\tau I(t \leqslant T) \nabla_\theta \mu_3\left(t, \mathbf{O}; \tilde{\boldsymbol{\theta}}, \tilde{\Lambda}_0\right) h_2(t) d\Lambda_0(t) \\
&\quad - \int_0^\tau I(t \leqslant T)\left\{\mu_3(t, \mathbf{O}; \boldsymbol{\theta}^*, \Lambda_0^*) h_2(t) + \mu_6(t, \mathbf{O}; \tilde{\boldsymbol{\theta}}, \tilde{\Lambda}_0)\int_t^T h_2(s) d\Lambda_0(s)\right\} d(\Lambda_0 - \Lambda_0^*)(t),
\end{aligned}
\tag{A.14}
$$

where $(\tilde{\boldsymbol{\theta}}, \tilde{\Lambda}_0) = \tilde{\varepsilon}(\boldsymbol{\theta}, \Lambda_0) + (1-\tilde{\varepsilon})(\boldsymbol{\theta}^*, \Lambda_0^*)$ for some $\tilde{\varepsilon} \in (0,1)$. Thus it follows that

$$\begin{aligned}
&\nabla S_{\psi^*}\left(\boldsymbol{\theta}-\boldsymbol{\theta}^*, \Lambda_0-\Lambda_0^*\right)[\mathbf{h}_1, h_2] \\
&= (\boldsymbol{\theta}-\boldsymbol{\theta}^*)^\top \mathbf{P}\left\{\nabla_\theta \mu_1\left(O;\boldsymbol{\theta}^*, \Lambda_0^*\right) - \int_0^T \nabla_\theta \mu_2\left(t, O; \boldsymbol{\theta}^*, \Lambda_0^*\right) d\Lambda_0^*(t)\right\}\mathbf{h}_1 \\
&\quad + \mathbf{h}_1^\top \int_0^\tau \mathbf{P}\Big[I(t \leqslant T)\Big\{\mu_4\left(t, O; \boldsymbol{\theta}^*, \Lambda_0^*\right) - \mu_2\left(t, O; \boldsymbol{\theta}^*, \Lambda_0^*\right) \\
&\qquad\qquad - \mu_5\left(t, \mathbf{O}; \boldsymbol{\theta}^*, \Lambda_0^*\right)\int_t^T d\Lambda_0(s)\Big\}\Big] d(\Lambda_0 - \Lambda_0^*)(t) \qquad \text{(A.15)} \\
&\quad - (\boldsymbol{\theta}-\boldsymbol{\theta}^*)^\top \int_0^\tau \mathbf{P}\left[I(t \leqslant T)\nabla_\theta \mu_3\left(t, \mathbf{O}; \boldsymbol{\theta}^*, \Lambda_0^*\right)\right] h_2(t) d\Lambda_0^*(t) \\
&\quad - \int_0^\tau \mathbf{P}\Big[I(t \leqslant T)\Big\{\mu_3\left(t, \mathbf{O}; \boldsymbol{\theta}^*, \Lambda_0^*\right) h_2(t) \\
&\qquad\qquad + \mu_6\left(t, \mathbf{O}; \boldsymbol{\theta}^*, \Lambda_0^*\right)\int_t^T h_2(s) d\Lambda_0^*(s)\Big\}\Big] d\left(\Lambda_0 - \Lambda_0^*\right)(t).
\end{aligned}$$

Since

$$\begin{aligned}
S(\psi)\left(\mathbf{h}_1, h_2\right) - S(\psi^*)\left(\mathbf{h}_1, h_2\right) =& \nabla S_{\psi^*}\left(\boldsymbol{\theta}-\boldsymbol{\theta}^*, \Lambda_0-\Lambda_0^*\right)[\mathbf{h}_1, h_2] \\
&+ o(\|\boldsymbol{\theta}-\boldsymbol{\theta}^*\| + \sup_{t\in[0,\tau]}|\Lambda_0(t)-\Lambda_0^*(t)|)\left(\|\mathbf{h}_1\| + \|h_2\|_V\right),
\end{aligned}$$

$S(\psi^*)$ is Frèchet differentiable.

Additionally, from (A.15) we find $\nabla S_{\psi^*}\left(\boldsymbol{\theta}-\boldsymbol{\theta}^*, \Lambda_0-\Lambda_0^*\right)[\mathbf{h}_1, h_2]$ can be rewritten as

$$\nabla S_{\psi^*}\left(\boldsymbol{\theta}-\boldsymbol{\theta}^*, \Lambda_0-\Lambda_0^*\right)[\mathbf{h}_1, h_2] = (\boldsymbol{\theta}-\boldsymbol{\theta}^*)^\top \Omega_1[\mathbf{h}_1, h_2] + \int_0^\tau \Omega_2[\mathbf{h}_1, h_2]\, d\left(\Lambda_0 - \Lambda_0^*\right), \qquad \text{(A.16)}$$

where

$$\begin{aligned}
\Omega_1[\mathbf{h}_1, h_2] =& \mathbf{h}_1^\top \mathbf{P}\left\{\nabla_\theta \mu_1\left(O;\boldsymbol{\theta}^*, \Lambda_0^*\right) - \int_0^T \nabla_\theta \mu_2\left(t, O; \boldsymbol{\theta}^*, \Lambda_0^*\right) d\Lambda_0^*(t)\right\} \\
&- \int_0^\tau \mathbf{P}\left[I(t \leqslant T)\nabla_\theta \mu_3\left(t, \mathbf{O}; \boldsymbol{\theta}^*, \Lambda_0^*\right)\right] h_2(t) d\Lambda_0^*(t),
\end{aligned}$$

and

$$\begin{aligned}
\Omega_2[\mathbf{h}_1, h_2] = & \mathbf{h}_1^\top \mathbf{P}\Big[I(t \leqslant T)\Big\{\mu_4\left(t, O; \boldsymbol{\theta}^*, \Lambda_0^*\right) - \mu_2\left(t, O; \boldsymbol{\theta}^*, \Lambda_0^*\right) \\
&\qquad - \mu_5\left(t, \mathbf{O}; \boldsymbol{\theta}^*, \Lambda_0^*\right)\int_t^T d\Lambda_0(s)\Big\}\Big] \\
&- \mathbf{P}\Big[I(t \leqslant T)\Big\{\mu_3\left(t, \mathbf{O}; \boldsymbol{\theta}^*, \Lambda_0^*\right) h_2(t) \\
&\qquad + \mu_6\left(t, \mathbf{O}; \boldsymbol{\theta}^*, \Lambda_0^*\right)\int_t^T h_2(s) d\Lambda_0^*(s)\Big\}\Big].
\end{aligned}$$

Obviously, $\Omega = (\Omega_1, \Omega_2)$ is a bounded linear operator from $\mathscr{H} \subset R^d \times BV[0,\tau]$ to $R^d \times BV[0,\tau]$ and $\nabla S_{\psi^*}\left(\boldsymbol{\theta} - \boldsymbol{\theta}^*, \Lambda_0 - \Lambda_0^*\right)[\mathbf{h}_1, h_2]$ can be treated as a bounded linear operator from $l^\infty(\mathscr{H})$ to itself by treating $(\boldsymbol{\theta} - \boldsymbol{\theta}^*, \Lambda_0 - \Lambda_0^*)$ as a element in $l^\infty(\mathscr{H})$ via the following definition

$$\left(\boldsymbol{\theta} - \boldsymbol{\theta}^*, \Lambda_0 - \Lambda_0^*\right)[\mathbf{h}_1, h_2] = (\boldsymbol{\theta} - \boldsymbol{\theta}^*)^\top \mathbf{h}_1 + \int_0^\tau h_2(t) d\left(\Lambda_0 - \Lambda_0^*\right)(t)$$

for any $(\mathbf{h}_1, h_2) \in R^d \times BV[0,\tau]$.

Next we will prove ∇S_{ψ^*} is invertible. To do that, it is sufficient to show that there exists some positive constant ε such that $\varepsilon\mathscr{H} \subset \Omega(\mathscr{H})$. Then we have

$$\begin{aligned}\|\nabla S_{\psi^*}(\Delta\theta, \Delta\Lambda_0)\|_{l^\infty(\mathscr{H})} &= \sup_{(\mathbf{h}_1, h_2)\in\mathscr{H}} \left| \Delta\boldsymbol{\theta}^T \Omega_1[\mathbf{h}_1, h_2] + \int_0^\tau \Omega_2[\mathbf{h}_1, h_2]\, d\Delta\Lambda_0(t) \right| \\ &= \|(\Delta\boldsymbol{\theta}, \Delta\Lambda_0)\|_{l^\infty(\Omega(\mathscr{H}))} \geqslant \varepsilon \|(\Delta\boldsymbol{\theta}, \Delta\Lambda_0)\|_{l^\infty(\mathscr{H})},\end{aligned}$$

which impies ∇S_{ψ^*} is invertible.

To prove $\varepsilon\mathscr{H} \subset \Omega(\mathscr{H})$ for some ε is equivalent to showing that Ω is one-to-one: if $\Omega[\mathbf{h}_1, h_2] = 0$, then $\mathbf{h}_1 = 0$ and $h_2 = 0$. To prove that, we choose $\boldsymbol{\theta} - \boldsymbol{\theta}^* = c\mathbf{h}_1$ and $\Lambda_0 - \Lambda_0^* = c\int h_2 d\Lambda_0^*$ in (A.16). Then we have $\nabla S_{\psi^*}(\mathbf{h}_1, h_2)[\mathbf{h}_1, h_2] = 0$ since $\Omega[\mathbf{h}_1, h_2] = 0$. Note that $\nabla S_{\psi^*}(\mathbf{h}_1, h_2)$ is the negative information matrix in the submodel $\left(\theta^* + \varepsilon\mathbf{h}_1, \Lambda_0^* + \int_0^t \varepsilon h_2 d\Lambda_0^*\right)$. Therefore, the score function for this submodel is zero with probability one. That is, with probability one, $\dot{l}_\theta(\boldsymbol{\theta}^*, \Lambda_0^*)^\top \mathbf{h}_1 + \dot{l}_{\Lambda_0}(\boldsymbol{\theta}^*, \Lambda_0^*)[h_2] = 0$. Under the assumptions specified in the beginning, we can get $\mathbf{h}_1 = 0$ and $h_2 = 0$.

On the other hand, using derivation similar to that in (A.14), we can verify that the class

$$\begin{Bmatrix}\dot{l}_\theta(\boldsymbol{\theta}, \Lambda_0)^\top \mathbf{h}_1 + \dot{l}_{\Lambda_0}(\boldsymbol{\theta}, \Lambda_0)[h_2] - \dot{l}_\theta(\boldsymbol{\theta}^*, \Lambda_0^*)^\top \mathbf{h}_1 - \dot{l}_{\Lambda_0}(\boldsymbol{\theta}^*, \Lambda_0^*)[h_2] : \\ \|\boldsymbol{\theta} - \boldsymbol{\theta}^*\| + \sup_{t\in[0,\tau]} |\Lambda_0(t) - \Lambda_0^*(t)| < \delta, (\mathbf{h}_1, h_2) \in \mathscr{H}\end{Bmatrix} \tag{A.17}$$

is P-Donsker for some $\delta > 0$ and that

$$\sup_{(\mathbf{h}_1, h_2)\in\mathscr{H}} \mathbf{P}\left[\dot{l}_\theta(\boldsymbol{\theta}, \Lambda_0)^\top \mathbf{h}_1 + \dot{l}_{\Lambda_0}(\boldsymbol{\theta}, \Lambda_0)[h_2] - \dot{l}_\theta(\boldsymbol{\theta}^*, \Lambda_0^*)^\top \mathbf{h}_1 - \dot{l}_{\Lambda_0}(\boldsymbol{\theta}^*, \Lambda_0^*)[h_2]\right]^2 \to 0 \tag{A.18}$$

as $\|\boldsymbol{\theta} - \boldsymbol{\theta}^*\| + \sup_{t\in[0,\tau]} |\Lambda_0(t) - \Lambda_0^*(t)| \to 0$. Then according to statement of Lemma 3.3.5 in Van Der Vaart and Wellner (1996), (A.17) and (A.18) combined with the consistency of $\hat{\psi}_m = (\hat{\boldsymbol{\theta}}, \hat{\Lambda}_0)$ yield

$$\left\|\mathbb{G}_m\left(\hat{\psi}_m - \psi^*\right)\right\|_{l^\infty(\mathscr{H})} = o_P\left(1 + \sqrt{m}\left\|\hat{\psi}_m - \psi^*\right\|_{l^\infty(\mathscr{H})}\right), \tag{A.19}$$

where $\mathbb{G}_m = \sqrt{m}\,(S_m - S)$. Therefore, by the definition of $\hat{\psi}_n$, we have

$$\begin{aligned}\sqrt{m}\left(S(\hat{\psi}_m) - S(\psi^*)\right)[\mathbf{h}_1, h_2] &= \sqrt{m}\left(S(\hat{\psi}_m) - S_m(\hat{\psi}_m)\right)[\mathbf{h}_1, h_2] \\ &= -\sqrt{m}\,(S_m - S)\,(\psi^*)\,[\mathbf{h}_1, h_2] + o_P\left(1 + \sqrt{m}\left\|\hat{\psi}_m - \psi^*\right\|_{l^\infty(\mathscr{H})}\right) \\ &= -\sqrt{m}\,(\mathbf{P}_m - \mathbf{P})\left\{\dot{l}_\theta(\boldsymbol{\theta}^*, \Lambda_0^*)^\top \mathbf{h}_1 + \dot{l}_{\Lambda_0}(\boldsymbol{\theta}^*, \Lambda_0^*)\,[h_2]\right\} + o_p(1),\end{aligned} \tag{A.20}$$

where the second equality holds due to (A.19). Since S is Frèchet differentiable at ψ^*, the left hand-side of (A.20) can be replaced by

$$\sqrt{m}\nabla S_{\psi^*}\left(\hat{\psi}_m - \psi^*\right)[\mathbf{h}_1, h_2] + o_P\left(\sqrt{m}\left\|\hat{\psi}_m - \psi^*\right\|_{l^\infty(\mathscr{H})}\right).$$

Performing $\nabla S_{\psi^*}^{-1}$ on both sides of (A.20) gives

$$\begin{aligned}&\sqrt{m}\left\{\left(\hat{\boldsymbol{\theta}} - \boldsymbol{\theta}^*\right)^\top \mathbf{h}_1 + \int_0^\tau h_2(t)d\left(\hat{\Lambda}_0 - \Lambda_0^*\right)(t)\right\} \\ &= -\sqrt{m}\,(\mathbf{P}_m - \mathbf{P})\left\{\dot{l}_\theta(\boldsymbol{\theta}^*, \Lambda_0^*)^\top \tilde{\mathbf{h}}_1 + \dot{l}_{\Lambda_0}(\boldsymbol{\theta}^*, \Lambda_0^*)\left[\tilde{h}_2\right]\right\} + o_p(1),\end{aligned}$$

where $(\tilde{\mathbf{h}}_1, \tilde{h}_2) = \Omega^{-1}(\mathbf{h}_1, h_2)$.

In particular, if we set $h_2 = 0$, then for any given $\mathbf{h}_1$, $\hat{\boldsymbol{\theta}}^\top \mathbf{h}_1$ is an asymptotic linear estimator for $\boldsymbol{\theta}^{*\top}\mathbf{h}_1$ with influence function $\dot{l}_\theta(\boldsymbol{\theta}^*, \Lambda_0^*)^\top \tilde{\mathbf{h}}_1 + \dot{l}_{\Lambda_0}(\boldsymbol{\theta}^*, \Lambda_0^*)\left[\tilde{h}_2\right]$ where $(\tilde{\mathbf{h}}_1, \tilde{h}_2) = \Omega^{-1}(\mathbf{h}_1, 0)$. Since this influence function falls into the linear space spanned by score functions, it is thus the efficient influence function. Therefore, $\hat{\boldsymbol{\theta}}$ is an estimator which attains semiparametric efficiency.

B ITERATION EXPRESSIONS IN EM

B.1 Expressions in M-step

The closed updating forms for ξ, σ^2 and D are given by

$$\xi^{(k+1)} = \left(\sum_{i=1}^m Z_i^\top Z_i\right)^{-1} \sum_{i=1}^m Z_i^\top \left[Y_i - X_i\eta^{(k+1)} - \mathrm{B}_i\left(\beta^{(k+1)} + \mathbb{E}_{i(k)}[b_i]\right)\right],$$

$$\begin{aligned}(\sigma^2)^{(k+1)} =& \frac{1}{N}\sum_{i=1}^m \Big\{ \left[Y_i - X_i\eta^{(k+1)} - Z_i\xi^{(k+1)} - \mathrm{B}_i\,\beta^{(k+1)}\right]^\top \Big[Y_i - X_i\eta^{(k+1)} - Z_i\xi^{(k+1)} \\ &- \mathrm{B}_i\left(\beta^{(k+1)} + 2\mathbb{E}_{i(k)}[b_i]\right)\Big] + \mathrm{tr}\left(Z_i^\top Z_i \mathbb{V}_{i(k)}[b_i]\right) + \mathbb{E}_{i(k)}[b_i]^\top Z_i^\top Z_i \mathbb{E}_{i(k)}[b_i]\Big\},\end{aligned}$$

and

$$D^{(k+1)} = \frac{1}{m}\sum_{i=1}^{m}\mathbb{E}_{i(k)}[b_i b_i^\top].$$

Recall that $r(b_i, t, \phi_1) = \exp\left\{\gamma^\top w_i + \alpha_1(x_i^\top \eta + \tilde{b}_i^\top B(t)) + \alpha_2\left(\tilde{b}_i^\top K(t_0, t)\tilde{b}_i\right)^{1/2}\right\}$ as defined in section 4.1. Let $a^{\otimes 2} = aa^\top$ for a column vector a, then the components of S_{ϕ_1} and I_{ϕ_1} can be finally expressed as follows,

$$S_\gamma = \sum_{i=1}^{m}\delta_i\left\{w_i - \frac{\sum_{j=1}^{m} Y_j(T_i)\mathbb{E}_{j(k)}\left[r(b_j, T_i, \theta)\right] w_j}{\sum_{j=1}^{m} Y_j(T_i)\mathbb{E}_{j(k)}\left[r(b_j, T_i, \theta)\right]}\right\},$$

$$I_\gamma = \sum_{i=1}^{m}\delta_i\left\{\frac{\sum_{j=1}^{m} Y_j(T_i)\mathbb{E}_{j(k)}\left[r(b_j, T_i, \theta)\right] w_j^{\otimes 2}}{\sum_{j=1}^{m} Y_j(T_i)\mathbb{E}_{j(k)}\left[r(b_j, T_i, \theta)\right]} - \frac{\left(\sum_{j=1}^{m} Y_j(T_i)\mathbb{E}_{j(k)}\left[r(b_j, T_i, \theta)\right] w_j\right)^{\otimes 2}}{\left(\sum_{j=1}^{m} Y_j(T_i)\mathbb{E}_{j(k)}\left[r(b_j, T_i, \theta)\right]\right)^2}\right\};$$

$$S_{\alpha_1} = \sum_{i=1}^{m}\delta_i\left\{x_i^\top \eta + B^\top(T_i)\left(\beta + \mathbb{E}_{i(k)}\left[b_i\right]\right) - \frac{\sum_{j=1}^{m} Y_j(T_i)\mathbb{E}_{j(k)}\left[r(b_j, T_i, \theta)(x_j^\top \eta + \tilde{b}_j^\top B(T_i))\right]}{\sum_{j=1}^{m} Y_j(T_i)\mathbb{E}_{j(k)}\left[r(b_j, T_i, \theta)\right]}\right\},$$

$$I_{\alpha_1} = \sum_{i=1}^{m}\delta_i\left\{\frac{\sum_{j=1}^{m} Y_j(T_i)\mathbb{E}_{j(k)}\left[r(b_j, T_i, \theta)\left(x_j^\top \eta + \tilde{b}_j^\top B(T_i)\right)^2\right]}{\sum_{j=1}^{m} Y_j(T_i)\mathbb{E}_{j(k)}\left[r(b_j, T_i, \theta)\right]} - \frac{\left(\sum_{j=1}^{m} Y_j(T_i)\mathbb{E}_{j(k)}\left[r(b_j, T_i, \theta)(x_j^\top \eta + \tilde{b}_j^\top B(T_i))\right]\right)^2}{\left(\sum_{j=1}^{m} Y_j(T_i)\mathbb{E}_{j(k)}\left[r(b_j, T_i, \theta)\right]\right)^2}\right\};$$

$$S_{\alpha_2} = \sum_{i=1}^{m}\delta_i\left\{\mathbb{E}_{i(k)}\left[\left(\tilde{b}_i^\top K(t_0, T_i)\tilde{b}_i\right)^{1/2}\right] - \frac{\sum_{j=1}^{m} Y_j(T_i)\mathbb{E}_{j(k)}\left[r(b_j, T_i, \theta)\left(\tilde{b}_j^\top K(t_0, T_i)\tilde{b}_j\right)^{1/2}\right]}{\sum_{j=1}^{m} Y_j(T_i)\mathbb{E}_{j(k)}\left[r(b_j, T_i, \theta)\right]}\right\},$$

$$I_{\alpha_2} = \sum_{i=1}^{m}\delta_i\left\{\frac{\sum_{j=1}^{m} Y_j(T_i)\mathbb{E}_{j(k)}\left[r(b_j, T_i, \theta)\left(\tilde{b}_j^\top K(t_0, T_i)\tilde{b}_j\right)\right]}{\sum_{j=1}^{m} Y_j(T_i)\mathbb{E}_{j(k)}\left[r(b_j, T_i, \theta)\right]} - \frac{\left(\sum_{j=1}^{m} Y_j(T_i)\mathbb{E}_{j(k)}\left[r(b_j, T_i, \theta)\left(\tilde{b}_j^\top K(t_0, T_i)\tilde{b}_j\right)^{1/2}\right]\right)^2}{\left(\sum_{j=1}^{m} Y_j(T_i)\mathbb{E}_{j(k)}\left[r(b_j, T_i, \theta)\right]\right)^2}\right\};$$

$$
\begin{aligned}
S_\beta =& \sum_{i=1}^{m} \delta_i \Bigg\{ \alpha_1 B(T_i) + \alpha_2 \mathbb{E}_{i(k)} \left[\left(\tilde{b}_i^\top K(t_0,T_i) \tilde{b}_i \right)^{-1/2} K(t_0,T_i)\tilde{b}_i \right] \\
& - \frac{\sum_{j=1}^m Y_j(T_i) \mathbb{E}_{j(k)} \left[r(b_j,T_i,\theta) \left(\alpha_1 B(T_i) + \alpha_2 \left(\tilde{b}_j^\top K(t_0,T_i)\tilde{b}_j \right)^{-1/2} K(t_0,T_i)\tilde{b}_j \right) \right]}{\sum_{j=1}^m Y_j(T_i)\mathbb{E}_{j(k)} \left[r(b_j,T_i,\theta) \right]} \Bigg\} \\
& + \frac{1}{\hat{\sigma}^2} \sum_{i=1}^m \mathrm{B}_i^\top \left(Y_i - X_i\eta - \mathrm{B}_i \left(\beta + \mathbb{E}_{i(k)}[b_i] \right) - Z_i \xi \right);
\end{aligned}
$$

$$
\begin{aligned}
S_\eta =& \sum_{i=1}^m \delta_i \left\{ \alpha_1 x_i - \frac{\sum_{j=1}^m Y_j(T_i)\mathbb{E}_{j(k)}\left[r(b_j,T_i,\theta) \right] \alpha_1 x_j}{\sum_{j=1}^m Y_j(T_i)\mathbb{E}_{j(k)}\left[r(b_j,T_i,\theta) \right]} \right\} \\
& + \frac{1}{\hat{\sigma}^2} \sum_{i=1}^m X_i^\top \left(Y_i - X_i\eta - \mathrm{B}_i(\beta + \mathbb{E}_{i(k)}[b_i]) - Z_i\xi \right), \\
I_\eta =& \sum_{i=1}^m \delta_i \left\{ \frac{\sum_{j=1}^m Y_j(T_i)\mathbb{E}_{j(k)}\left[r(b_j,T_i,\theta) \right] \alpha_1^2 x_j^{\otimes 2}}{\sum_{j=1}^m Y_j(T_i)\mathbb{E}_{j(k)}\left[r(b_j,T_i,\theta) \right]} - \frac{\left(\sum_{j=1}^m Y_j(T_i)\mathbb{E}_{j(k)}\left[r(b_j,T_i,\theta) \right] \alpha_1 x_j \right)^{\otimes 2}}{\left(\sum_{j=1}^m Y_j(T_i)\mathbb{E}_{j(k)}\left[r(b_j,T_i,\theta) \right] \right)^2} \right\} \\
& + \frac{1}{\hat{\sigma}^2} \sum_{i=1}^m X_i^\top X_i - (\hat{\sigma}^2)^{-2} \frac{2}{N} \sum_{i=1}^m \left(X_i^\top \left[Y_i - X_i\eta - \mathrm{B}_i\left(\beta + \mathbb{E}_{i(k)}[b_i] \right) - Z_i\xi \right] \right)^{\otimes 2}.
\end{aligned}
$$

B.2 Expressions in E-step

To get $\hat{b}_i := \arg\max_{b_i} \log p(T_i, \delta_i, Y_i, b_i; \boldsymbol{\theta}^{(k)}, \Lambda_0^{(k)})$ in E-step, the Newton-Raphson method performs as follows:

$$
\hat{b}_i^{\text{it}+1} = \hat{b}_i^{\text{it}} + \mathcal{I}_i^{-1} S_{b_i} \mid_{b_i = \hat{b}_i^{\text{it}}},
$$

where 'it' denotes the iteration counter, and

$$
\begin{aligned}
S_{b_i} =& \frac{\partial \log p\left(T_i, \delta_i, Y_i, b_i; \theta^{(k)} \right)}{\partial b_i} \\
=& \delta_i \left\{ \alpha_1^{(k)} B(T_i) + \alpha_2^{(k)} \left[\left(\beta^{(k)} + b_i \right)^\top K(t_0,T_i) \left(\beta^{(k)} + b_i \right) \right]^{-1/2} K(t_0,T_i) \left(\beta^{(k)} + b_i \right) \right\} \\
& - \sum_{T_j \leqslant T_i} \Lambda_0^{(k)}\{T_j\} r(b_i, T_j, \theta^{(k)}) \Bigg\{ \alpha_1^{(k)} B(T_j) \\
& \qquad + \alpha_2^{(k)} \left[\left(\beta^{(k)} + b_i \right)^\top K(t_0,T_j) \left(\beta^{(k)} + b_i \right) \right]^{-1/2} K(t_0,T_j) \left(\beta^{(k)} + b_i \right) \Bigg\} \\
& + \frac{\mathrm{B}_i^\top \left(Y_i - X_i \eta^{(k)} - \mathrm{B}_i \left(\beta^{(k)} + b_i \right) - Z_i \xi^{(k)} \right)}{(\sigma^2)^{(k)}} - (D^{(k)})^{-1} b_i.
\end{aligned}
$$

C SIMULATION STUDIES

C.1. Case 2: Simulation study based on the MRC trial

To mimic the MRC trial, simulation data in Case 2 is generated as follows:

- Set sample size $m = 3700$ and $t_{ij} = (0, 0.041, 0.082, 0.166, 0.25, 0.5, 0.75, 1, ..., 5.75)$ with fixed difference 0.25 from 1 to 5.75. Baseline covariates in the longitudinal model are specified as $x_i = (x_{1i}, x_{2i}, x_{3i})^\top$, where x_{1i} and x_{2i} are binary covariates (corresponding to sex and smoke indicator in MRC) generated independently from Bernoulli distribution with probability 0.42 and 0.164 respectively; x_{3i} is generated from truncated normal distribution with mean 7, variance 0.29 and truncated interval $[6.5, 7.4]$ (denoted as TN$(7, 0.29; [6.5, 7.4])$ in the following) to mimic the shape of ages (divided by 10) in the MRC trial. The external stimulus, z_{ij}, is specified as a single covariate taking value 1 if t_{ij} falls into the set $\{0, 1, 2, 3, 4, 5\}$ and 0 otherwise. Cubic B-spline bases are constructed with two interior knots located at 0.25 and 1.5 (q=6). b_i is of $q - 1$ dimension rather than q because the variance of b_{1i} is very close to 0 as suggested by the MRC trial data analysis.

- Baseline covariates $w_i = (w_{1i}, w_{2i}, x_i^\top)^\top$ in the survival model is of five dimension where $(w_{1i}, w_{2i}, 1-w_{1i}-w_{2i})$, corresponding to the treatment indicator in the MRC trial, is generated from multinomial distribution with parameters $n = 1$ and $p = (0.25, 0.25, 0.5)$. (w_{1i}, w_{2i}) also makes an impact on the longitudinal process via interaction terms between spline bases and treatments in fixed-effects part. $\beta_k^{(1)}$ and $\beta_k^{(2)}$ denote the effects of interaction terms $w_{1i} * B_k(t)$ and $w_{2i} * B_k(t)$ respectively, for $k = 1, ..., q$.

- $\lambda_0(t) = \exp\{0.7(t-1.5)\}$ if $t > 1.5$ and 0 otherwise. Censoring times are generated from a mixture distribution composed of TN$(3, 1; [1.5, 5.8])$ and TN$(5.2, 0.3^2; [1.5, 5.8])$ by mixing weights 0.6 and 0.4 respectively, leading to approximately 90% censoring.

C.2. Case 3: Simulation study for a nonparametric setting

The candidate knot patterns (the number and location of interior knots) we considered in case 3 are given below:

- Pattern 1: 0.5-quantile of observation times;
- Pattern 2: 0.75-quantile of observation times;
- Pattern 3: (0.5, 0.75)-quantiles of observation times;
- Pattern 4: (0.25, 0.5, 0.75)-quantiles of observation times;
- Pattern 5: (0.33, 0.66)-quantiles of observation times;
- Pattern 6: 3, i.e., the mid-time of the follow-up interval

Pattern 6 is selected as the best pattern based on AIC and BIC in each Monte Carlo replications. Among 500 Monte Carlo replications, AIC and BIC always give consistent selection results. In addition to the estimation results of survival parameters shown in Table 4 in the main article, we evaluate the estimation of longitudinal trajectories by mean squared error (MSE)

$$\text{MSE} = \frac{1}{N}\sum_{i=1}^{m}\sum_{j=1}^{n_i}\{\hat{m}_i(t_{ij}) - m_i(t_{ij})\}^2,$$

where $\hat{m}_i(t) = \sum_{k=1}^{q}(\hat{\beta}_k + \hat{b}_{ik})B_k(t)$ and $m_i(t) = \nu_{1i}\{(-1/6)(t-3)^3 + (t-3)\} + \nu_{2i}$. The average (SD) values of MSE for JM, TS and No Variability are 0.0511 (0.00125), 0.0517 (0.00126) and 0.0518 (0.00126), respectively.

C.3. Case 4: Simulation study for a sine function setting

In case 4, we set the true longitudinal trajectory for the i-th individual as $m_i(t) = \nu_{1i}\sin(t) + \nu_{2i}$ for $i = 1, ..., 1000$, where a_{1i} and a_{2i} are random parameters generated from $U(0.5, 2)$ and $U(2, 5)$, respectively. Measurement times are specified as $t_{ij} = \{0, 0.4, 0.8, 1.2, 1.6, 2, 2.5, ...6\}$ (every half year after 2 years)

during the follow-up interval $[0, 6]$. The baseline hazard function $\lambda_0(t)$ is specified as $\exp\{-2.5\}$ if $t > 2$ and 0 otherwise. Other parameters are specified in a similar manner to case 3.

The candidate knot patterns (the number and location of interior knots) we considered in case 4 are given below:

- Pattern 1: $\pi/2$, $3\pi/2$;
- Pattern 2: π;
- Pattern 3: $\pi/2$,π,$3\pi/2$;
- Pattern 4: 0.5-quantile of observation times;
- Pattern 5: 0.75-quantile of observation times;
- Pattern 6: (0.25,0.5,0.75)-quantiles of observation times;
- Pattern 7: 3, i.e., the mid-time of the follow-up interval

Table S1 shows the selection results of knot patterns among 500 Monte Carlo simulations. Nearly 60% of simulations chose Pattern 2 (one knot: π) and 40% chose Pattern 7 (one knot: 3) and the two patterns are very similar. As can be seen from Table S2, the performances between JM and TS are similar in this case since the approximation via a spline regression function to a sine function is the dominant source of bias. The association parameter α_2, compared with α_1, is estimated with larger bias under JM and TS. Note that the second derivative of a sine function is still a sine function, whereas it is approximated by a linear spline function in JM and TS. Similar to the result in case 3, the comparison between JM (or TS) and No Variability shows that taking the biomarker variability into account benefits the estimation of γ and α_1.

Table S1. Selected knot pattern among 500 simulations

Pattern 2	Pattern 5	Pattern 7
293	2	205

Table S2. Simulation results in case 4. SD is the MC standard deviation of the estimates across simulated data sets; CP is the 95% coverage probability.

	JM			TS			No Variability			True model		
True	$\hat{\theta}$	SD	CP	$\hat{\theta}$	SD	CP	$\hat{\theta}$	SD	CP	$\hat{\theta}$	SD	CP
$\gamma = -1$	−0.9901	0.0736	0.948	−0.9840	0.0730	0.944	−0.9556	0.0749	0.842	−0.9973	0.0731	0.948
$\alpha_1 = 0.3$	0.3089	0.0428	0.940	0.3121	0.0427	0.930	0.3877	0.0391	0.366	0.3022	0.0385	0.948
$\alpha_2 = 0.3$	0.2235	0.0350	0.622	0.2206	0.0334	0.610	-	-	-	0.2993	0.0431	0.950

References

TSIATIS, A. A. (1981). A large sample study of cox's regression model. *The Annals of Statistics* **9**(1), 93–108.

VAN DER VAART, A. AND WELLNER, J. A. (1996). Weak convergence. In: *Weak convergence and empirical processes*. Springer, pp. 16–28.

ZENG, D. AND CAI, J. (2005). Asymptotic results for maximum likelihood estimators in joint analysis of repeated measurements and survival time. *The Annals of Statistics* **33**(5), 2132–2163.

[*Received XXX; revised XXX; accepted for publication XXX*]
